# Diagnostic Accuracy of Artificial Intelligence Based on Imaging Data for Preoperative Prediction of Microvascular Invasion in Hepatocellular Carcinoma: A Systematic Review and Meta-Analysis

**DOI:** 10.3389/fonc.2022.763842

**Published:** 2022-02-24

**Authors:** Jian Zhang, Shenglan Huang, Yongkang Xu, Jianbing Wu

**Affiliations:** ^1^ Department of Oncology, The Second Affiliated Hospital of Nanchang University, Nanchang, China; ^2^ Department of Digestive Oncology, Jiangxi Key Laboratory of Clinical and Translational Cancer Research, Nanchang, China

**Keywords:** hepatocellular carcinoma, artificial intelligence, deep learning, machine learning, microvascular invasion (MVI), radiomics

## Abstract

**Background:**

The presence of microvascular invasion (MVI) is considered an independent prognostic factor associated with early recurrence and poor survival in hepatocellular carcinoma (HCC) patients after resection. Artificial intelligence (AI), mainly consisting of non-deep learning algorithms (NDLAs) and deep learning algorithms (DLAs), has been widely used for MVI prediction in medical imaging.

**Aim:**

To assess the diagnostic accuracy of AI algorithms for non-invasive, preoperative prediction of MVI based on imaging data.

**Methods:**

Original studies reporting AI algorithms for non-invasive, preoperative prediction of MVI based on quantitative imaging data were identified in the databases PubMed, Embase, and Web of Science. The quality of the included studies was assessed using the Quality Assessment of Diagnostic Accuracy Studies 2 (QUADAS-2) scale. The pooled sensitivity, specificity, positive likelihood ratio (PLR), and negative likelihood ratio (NLR) were calculated using a random-effects model with 95% CIs. A summary receiver operating characteristic curve and the area under the curve (AUC) were generated to assess the diagnostic accuracy of the deep learning and non-deep learning models. In the non-deep learning group, we further performed meta-regression and subgroup analyses to identify the source of heterogeneity.

**Results:**

Data from 16 included studies with 4,759 cases were available for meta-analysis. Four studies on deep learning models, 12 studies on non-deep learning models, and two studies compared the efficiency of the two types. For predictive performance of deep learning models, the pooled sensitivity, specificity, PLR, NLR, and AUC values were 0.84 [0.75–0.90], 0.84 [0.77–0.89], 5.14 [3.53–7.48], 0.2 [0.12–0.31], and 0.90 [0.87–0.93]; and for non-deep learning models, they were 0.77 [0.71–0.82], 0.77 [0.73–0.80], 3.30 [2.83–3.84], 0.30 [0.24–0.38], and 0.82 [0.79–0.85], respectively. Subgroup analyses showed a significant difference between the single tumor subgroup and the multiple tumor subgroup in the pooled sensitivity, NLR, and AUC.

**Conclusion:**

This meta-analysis demonstrates the high diagnostic accuracy of non-deep learning and deep learning methods for MVI status prediction and their promising potential for clinical decision-making. Deep learning models perform better than non-deep learning models in terms of the accuracy of MVI prediction, methodology, and cost-effectiveness.

**Systematic Review Registration:**

https://www.crd.york.ac.uk/PROSPERO/display_record.php? RecordID=260891, ID:CRD42021260891.

## Introduction

Hepatocellular carcinoma (HCC) is the most common primary liver malignancy and the fourth most common cause of cancer-related deaths worldwide (1). Liver transplantation and resection are the only potentially curative treatments (2). However, a high risk of recurrence and metastasis after resection leads to a poor prognosis for patients with HCC (3). HCC is highly heterogeneous at the histological, molecular, and genetic levels, making its prognostic stratification and personalized management challenging.

The presence of microvascular invasion (MVI) is considered an independent prognostic factor associated with HCC’s early recurrence and poor survival after resection. For MVI-positive patients, expanding resection margins can distinctly improve patient survival by eradicating micrometastases (4, 5). In the current era of precision medicine, a proportion of patients in each stage do not fulfill the criteria for the treatment’s allocation (6). In a recent article, Li et al. reported that surgical resection, rather than ablation, is more effective in treating small HCC with MVI. For the MVI patients, cumulative early recurrence rates were significantly lower in the surgical resection group than in the radiofrequency ablation group (22.8% *vs.* 52.5% after 1 year; 30.6% *vs.* 90.0% after 2 years) (7, 8). For HCC patients with MVI present, a more aggressive treatment strategy may be preferred, such as expanding the resection margin or anatomical resectioning for patients undergoing hepatic resectioning, minimizing the ablation margin to at least 0.5–1 cm for patients receiving ablation, and neoadjuvant therapy before surgery (9, 10). Hence, to better allocate treatment strategies, predicting the risk of early recurrence of HCC before resection or ablation is crucial. MVI is not similar to macrovascular invasion, which can be evaluated using radiologic images. MVI is defined as the presence of a tumor in either the portal, hepatic venous system or the branches surrounding the hepatic tissue lined by endothelium, which is visible only by microscopy (11). Many studies have shown that MVI is directly related to the outcomes of HCC patients after surgery, and many researchers have attempted to predict MVI using preoperative imaging analysis.

Recently, in the medical imaging domain, radiomics features extracted through non-deep learning (NDL) algorithms (NDLAs) have been proposed, which are effective for predicting MVI (12). Moreover, artificial intelligence (AI) algorithms have been widely applied in the classification of skin cancer (13), diagnosis of eye diseases (14), identification of prostate cancer (15), and brain metastasis detection (16). AI algorithms show promising performance in the imaging diagnosis of liver cancer (17–20).

Radiomics is a high-throughput extraction of large amounts of quantitative imaging features with the assistance of NDLAs (12). However, manual feature extraction is complicated and time-consuming and lacks stability and consistent interpretation (21). Compared with the NDL used by radiomics analysis, deep learning (DL) algorithms (DLAs) have an advantage in learning features from the images directly, rather than using artificially defined features by human experience (22–24). DL in medical imaging analysis has two properties: multiple layers of non-linear processing units and supervised or unsupervised learning of feature presentations on each layer (23). Input data for DLAs consist of the imaging data itself such as different CT and MRI sequence sets, whereas output data are the desired parameters that should be extracted from the imaging data. In general, the dataset is usually randomly divided into training and testing sets. The former is used to train the DL model; the DLAs attempt to calculate the complex relationship between input and output data. The latter is then used to test the performance of the DL model on a new dataset that had not been utilized to train the DL model.

Recently, some reports have utilized DL methods based on imaging data [MRI, CT, and ultrasound (US)] to predict MVI with satisfactory performance. However, these reports were limited to a small sample size. Huang et al. performed a meta-analysis of radiomics and non-radiomics methods based on medical image data for MVI prediction (25). Currently, there is no systematic review or meta-analysis of DL methods concerning MVI prediction for HCC patients. In addition, studies comparing DL and NDL methods for MVI prediction are rare. Hence, to provide a more comprehensive and expansive summary of these studies and further recognize the prediction performance of DL for MVI prediction, we conducted a systematic review and meta-analysis by comparing the performance of DL and NDL methods for MVI prediction.

Therefore, the objective of this systematic review and meta-analysis was to assess DL and NDL concerning MVI prediction and compare their performances.

## Materials and Methods

This systemic review and meta-analysis was conducted in accordance with the Preferred Reporting Items for Systematic Reviews and Meta-Analyses (PRISMA) statement recommended by the Cochrane Collaboration. This study was prospectively registered in PROSPERO (ID: CRD42021260891).

### Search Strategy

Papers describing the use of AI, NDL, and DL for the prediction of HCC were reviewed. We searched the PubMed and Web of Science databases. All English publications until June 14, 2021, were searched without any restrictions on countries or article types. Search terms are available in the **Supplementary Search Strategy** and were included when they discussed the use of NDL or DL methodologies on images in MVI prediction.

### Eligibility Criteria

After the removal of duplicates, the articles were reviewed to identify studies that satisfied the following criteria: 1) population: pathologically confirmed HCC patients after surgical resection; 2) intervention: evaluation of MVI using AI algorithms based on quantitative imaging data preoperatively; 3) outcome: diagnostic accuracy of imaging analysis for diagnosing or predicting MVI in HCC study; and 4) design: any type of study design, including observational studies (retrospective or prospective) and clinical trials. Studies were excluded according to the following criteria: 1) studies with duplicate patients and data; 2) case reports, review articles, letters, conference abstracts, and editorials; and 3) studies not in the field of interest. All identified articles were first screened by title and abstract, and then full-text reviews of potentially eligible articles were performed.

### Data Extraction

The following information was extracted from the eligible articles: a) study characteristics: authors (years of publication), study type, study design, and study location; b) subject characteristics: operation, interval image exam, number of tumors, etiology of HCC [the number of hepatitis B virus (HBV) or hepatitis C virus (HCV)], tumor size, the numbers of MVI-present and MVI-absent, variables with p < 0.05 between MVI(+) and MVI(−), and variables with p < 0.05 between the training and testing sets; c) model characteristics: image, region segmentation, validation method, input data, feature selection, and modeling method; and d) the performance of the DL or NDL model: the area under the curve (AUC) value and the numbers of true positives (TP), false positives (FP), false negatives (FN), and true negatives (TN). The reference formulas were as follows: sensitivity = TP/(TP + FN) and specificity = TN/(FP + TN). If there was no sensitivity or specificity in one study, we used Engauge Digitizer version 12.1 to calculate sensitivity and specificity when Youden’s index was max based on the receiver operating characteristic (ROC) curve in articles. If there were more than two models in the same group of patients in one study, the model with a higher AUC value was included in our meta-analysis. If some models only analyzed imaging data and others that analyzed both imaging data and clinical parameters, then only the former were included in this study.

### Assessment of Study Quality

Two reviewers independently assessed the quality of the eligible articles using the Quality Assessment of Diagnostic Accuracy Studies 2 (QUADAS-2) criteria and the four domains of patient selection, index test, reference standard, and flow of patients through the study (26).

### Data Synthesis and Statistical Analysis

The pooled sensitivity, specificity, positive likelihood ratio (PLR), negative likelihood ratio (NLR), and AUC value of the receiver operating curve were computed. The results are shown in a forest plot. The presence of a threshold effect was analyzed by calculating Spearman’s correlation coefficient between sensitivity and the false-positive rate (when p < 0.05, the threshold was defined as present). When substantial heterogeneity was noted, a meta-regression analysis was performed to identify the causes. The random-effects model was used to calculate the meta-analytic pooled AUC value, and Higgins’s I^2^ test was used to assess the heterogeneity between included studies with I^2^ > 75% deemed considerable heterogeneity. An influence analysis was performed if I^2^ > 90%. For all NDL and DL models, excluding models using US, to determine the source of heterogeneity, meta-regression analysis based on the number of tumors (single or multiple), image (CT or MRI), region segmentation (manual or semiautomatic), set (validation or training set), least absolute shrinkage and selection operator (LASSO), support vector machine (SVM), convolutional neural network (CNN), 3D-CNN, arterial phase (AP), and portal venous phase (PVP) sequence was performed. For all NDL models excluding US, meta-regression analysis based on the number of tumors, image, region segmentation, set, LASSO, and SVM was performed.

Publication bias was evaluated using Deeks’ funnel plot and Deeks’ asymmetry test. The AUC values of 0.5–0.7, 0.7–0.9, and >0.9 indicate low, moderate, and high diagnostic power, respectively. All statistical analyses were conducted using STATA version 14.0 (StataCorp LP, College Station, TX, USA) and Meta-DiSc version 1.4.

## Results

### Search Results and Qualitative Assessment

**Figure 1:**
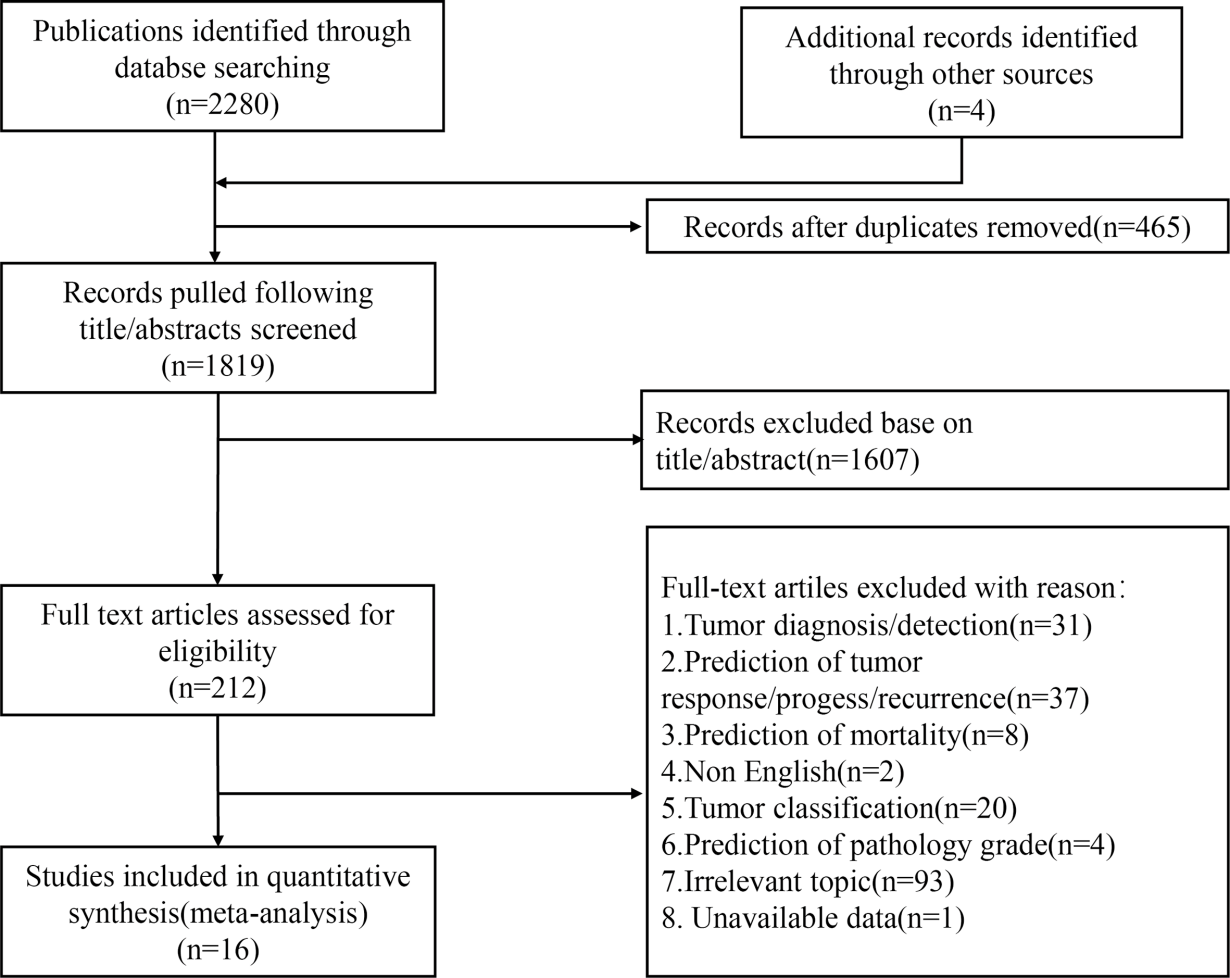
Flowchart of study selection.

The PRISMA flow diagram systematically depicts the study selection process (**Figure 1**). A total of 2,280 publications and four articles identified through a meta-analysis were initially retrieved through literature searches, 1,819 of them remaining after the removal of duplicates. After title and abstract screening, 212 articles reported the use of AI in HCC. After a full-text assessment, 16 studies were included in the systematic review and meta-analysis. The quality of the included studies was assessed using the QUADAS-2 scale (26). The results of the qualitative assessment of the included studies are shown in **Supplementary Figure S1**.

### Review of the Included Studies

**Tables 1**, **2** present the detailed characteristics of the 16 studies. Fifteen of the studies were single-center and retrospective studies that used an internal validation method (random splitting or cross-validation) to assess the performance of the MVI prediction model. One study was multicentered and retrospective and used an external validation method. All patients were diagnosed with HCC based on postoperative pathologic specimens and had available preoperative imaging data including CT, MRI, or US. Fifteen studies were based on a population from China 4 (27–41) and one from the United States (42). Concerning the etiology of HCC, at least 78.46% of patients had HBV or HCV of 4,657 patients across all included studies. In patient selection, five articles only included HCC patients with single tumors and excluded multiple tumors (27, 34, 39–41). Based on this diagnostic meta-analysis, 1,946 (40.89%) patients were pathologically diagnosed as MVI-present and 2,813 patients as MVI-absent after surgical resection or liver transplantation. In addition to tumor size in the study by Feng et al. and the hypodense halo in the study by Jiang et al., no significant differences in clinical variables were observed between the training and validation groups. Other characteristics of the included studies are presented in **Tables 1**, **2**, and the baseline characteristics of this meta-analysis are presented in **Table S1**.

**Table 1:** Characteristics of the included studies.

Authors (year of publication)	Study type	Study design	Study location	Operation	Interval image exam	Number of tumors	Validation	Image	Region segmentation	Input data	Feature selection	Modeling method
Song et al. (2021) (27)	Retro.	Single center	China	SR	Within 1 month	Single	Randomly split at a ratio	MRI	Manually drawn	ADC, DWI (b = 0), DWI (b = 500), AP, PVP, DP, T1WI, T2WI	NA	Radiomics model, CNN
Jiang et al. (2021) (28)	Retro.	Single center	China	SR or TL	Within 2 months	Multiple	Randomly split at a ratio	CT	Manually drawn with ITK-SNAP software	AP, PVP, and DP	NA	XGBoost, 3D-CNN
Wang et al. (2020) (29)	Retro.	Single center	China	SR	Unclear	Multiple	Randomly split at a ratio	MRI	Manually drawn	DWI (b0, b100, b600, and ADC images)	CNN	CNN with DSN
Zhou et al. (2021) (30)	Retro.	Single center	China	SR	Within 1 month	Multiple	Randomly split at a ratio	Gd-EOB-DTPA-enhanced MRI	Manually drawn	Pre-contrast, AP, PVP	3D-CNN	3D-CNN with DSN
Zhang et al. (2021) (31)	Retro.	Single center	China	SR	Within 1 week	Multiple	Randomly split at a ratio	MRI	Manually drawn with ITK-SNAP software	T2WI, T2-SPIR, and PVP images	3D-CNN	3D-CNN
Wei et al. (2021) (32)	T: Retro. V: Pro.	Multicenter	China	SR	Within 1 month	Multiple	External validation	MRI	Manually drawn	CT: AP, PVP MRI: T2W1, T1WI, AP, PVP, HBP	CNN	CNN

Retro, retrospective; Pro, prospective; CNN, convolutional neural network; AP, arterial phase; PVP, portal venous phase; DP, delayed phase; DSN, deep supervision network; V, validation set; T, training set; SR, surgical resection; TL, liver transplantation; LASSO, least absolute shrinkage and selection operation; SVM, support vector machine; BPNet, back-propagation neural network; KNN, k-nearest neighbors; RF, random forest; DT, decision tree; GBDT, gradient boosting decision tree; NRS, neighborhood rough set; PCA, principal component analysis; XGBoost, extreme gradient boosting; ADC, apparent diffusion coefficient.

NA, not available.

**Table 2:** Characteristics of the included studies.

Authors (year of publication)	Study type	Study design	Study location	Operation	Interval image exam	Number of tumors	Validation	Image	Region segmentation	Input data	Feature selection	Modeling Method
Feng et al. (2019) (33)	Retro.	Single center	China	SR	Within 1 month	Multiple	Randomly split at a ratio	Gd-EOB-DTPA-enhanced MRI	Manually drawn with ITK-Snap software	T1WI in/out phase, T1WI-FS, T1WI+c, T2WI+c, T1WI (HBP)	LASSO	LASSO regression model
Nebbia et al. (2020) (42)	Retro.	Single center	USA	SR	Within a week	Multiple	Stratified 5-fold cross-validation	MRI	Manually drawn	DWI, T1, T2, late AP, and PVP	LASSO, feature stability analysis	SVM, decision trees, KNN, Bayes
Liu et al. (2021) (34)	Retro.	Single center	China	SR	Unclear	Single	Randomly split at a ratio	CT	Manually drawn with 3D-Slice software	AP	Intraclass correlation coefficient, LASSO	logistics regression
Dong et al. (2020) (35)	Retro.	Single center	China	SR	Within 2 weeks	Multiple	Split at a ratio	Ultrasound	Manually drawn with MITK	NA	Pearson correlation analysis, minimum redundancy maximum relevance	RF
Xu et al. (2019) (36)	Retro.	Single center	China	SR or TL n (n = 16)	Unclear	Multiple	Split at a ratio	CT	Semiautomatically drawn with Python	AP, PVP	recursive feature selection SVM, step-wise multivariate analysis	Ref-SVM, multivariate regression
Hu et al. (2018) (40)	Retro.	Single center	China	SR	Within 2 weeks	Single	Split at a ratio	Ultrasound	Manually drawn with the A.K. software	NA	LASSO	Logistic regression
Yao et al. (2018) (37)	Retro.	Single center	China	SR	Unclear	Unclear	Cross-validation	Ultrasound	Manually drawn	NA	Sparse representation	SVM
Ni et al. (2019) (38)	Retro.	Single center	China	SR or TL	Within 1 month	Unclear	Split at a ratio	CT	Manually drawn with the A.K. software	PVP	LASSO, NRS, PCA	BPNet, KNN, SVM, RF, DT, Bayes, GBDT
Peng et al. (2018) (39)	Retro.	Single center	China	SR	Within 1 week	Single	Split at a ratio	CT	Semiautomatically drawn with MATLAB	AP, PVP	LASSO	logistic model
Ma et al. (2018) (41)	Retro.	Single center	China	SR	Unclear	Single	Split at a ratio	CT	Manually drawn with ITK-SNAP software	AP, PVP, DP	LASSO	SVM

Retro, retrospective; AP, arterial phase; PVP, portal venous phase; DP, delayed phase; SR, surgical resection; TL, liver transplantation; LASSO, least absolute shrinkage and selection operation; SVM, support vector machine; BPNet, back-propagation neural network; KNN, k-nearest neighbors; RF, random forest; DT, decision tree; GBDT, gradient boosting decision tree; NRS, neighborhood rough set; PCA, principal component analysis.

NA, not available.

Chen et al. compared the predictive performance of five classifiers in six different MRI sequences, and the analysis showed that SVM, extreme gradient boosting (XGBoost), and logistic regression (LR) classifiers in the validation cohort showed greater diagnostic efficiency for predicting MVI and NDL models based on delayed hepatobiliary phase (HBP). Due to a lack of data, the study by Chen et al. was excluded from this meta-analysis.

In the study by Nebbia et al., the imaging data were artificially defined as the margin and tumor region before they were used for training models. The results showed that the model combined with margin radiomics and tumor radiomics performed generally worse than single tumor radiomics, contradicting the conclusions of Feng et al. (33). The probable causes included the small sample size, and the tumor margin region may have included extrahepatic regions in the margin segmentation process. Another important reason is that features of the model that combine with margin radiomics and tumor radiomics must be features from both margin and tumor regions, preventing some predictive value features from being learned. In addition, Xu et al. found that analyzing radiomics features from peritumoral regions to calculate predictive performance is not superior to using features from the intratumoral region.

Owing to the high dimensionality and complexity of imaging data using different sequences, feature selection was used to reduce the computational power required to conduct such complex analyses. The LASSO was frequently used for feature selection (33, 34, 38–42). Other methods, which were frequently used for classification, include LASSO regression (33, 34, 40, 43), SVMs (32, 36, 38, 41), decision trees (27), k-nearest neighbor (30, 32), XGBoost (30, 33), and random forest (30, 35).

In contrast to NDL, feature selection and classification of DL occur simultaneously in the process of classifier training. Six of the included studies reported the DL method for the prediction of MVI. **Table S2** summarizes the details of these six studies. Three of the included studies, each a CNN, was used to build the MVI prediction model (27, 29, 32). In three of the included studies, the 3D-CNN model was developed to assess MVI in an end-to-end training fashion, in which feature extraction and predictive model construction were automatically processed by a single neural network (28, 30, 31). While training the DL model, Wu et al. and Wang et al. proposed a deep supervision network (DSN) to reduce the loss function and improve the performance of the DL model by directly supervising the features of the hidden layer and improving the effectiveness of the hidden layer during the CNN learning process (29, 30).

It is worth mentioning that Song et al. proposed a CNN model through MRI analysis of 601 HCC patients with single tumors and then compared the performances of the CNN model and radiomics model based on the same group. The results showed that the CNN model achieved an AUC of 0.915 (0.868–0.963) in the testing cohort as compared to the radiomics model with an AUC of 0.731 (0.645–0.817). In addition, survival analysis demonstrated that patients with DLC-predicted MVI status were associated with poor overall survival and recurrence-free survival, suggesting the strong clinical value of the DLC model in preoperatively identifying HCC patients with poor prognosis and guiding the resection range. Similarly, through CT imaging analysis of 405 HCC patients, Jiang et al. proposed and compared the 3D-CNN model, radiomics model, radiological model, and RRC model (model combining radiological features, radiomics features, and clinical variables), with the results showing that the DL model achieved the highest AUC of 0.906 in the validation set. Survival analysis showed that recurrence-free survival was significantly better in the predicted MVI-negative group than in the predicted MVI-positive group. Furthermore, in one multicenter retrospective study, 750 HCCs were enrolled from five Chinese hospitals, and a CNN model (n = 309) based on CT imaging analysis and another (n = 329) based on MRI analysis were trained. In the external validation cohort (n = 115), the findings revealed that the MRI-based CNN model achieved superior prediction performance (AUC: 0.812 *vs.* 0.736, p = 0.038; sensitivity: 70.4% *vs.* 57.4%, p = 0.015; specificity: 80.3% *vs.* 86.9%, p = 0.052). Survival analysis showed that both DL models could stratify groups with both high and low risk in terms of progression-free survival and overall survival. From the three studies, the high diagnostic power of the CNN model was validated, and consistent results indicated the potential value in clinical decision-making.

### Meta-Analysis of the Included Studies

In total, 18 NDL models and 11 DL models with 4,759 cases described in 16 individual studies were retrieved. Meta-analysis was performed separately in the subgroups for different modeling methods in different cohorts.

### Deep Learning Model for Preoperative Microvascular Invasion Evaluation

**Figure 2:**
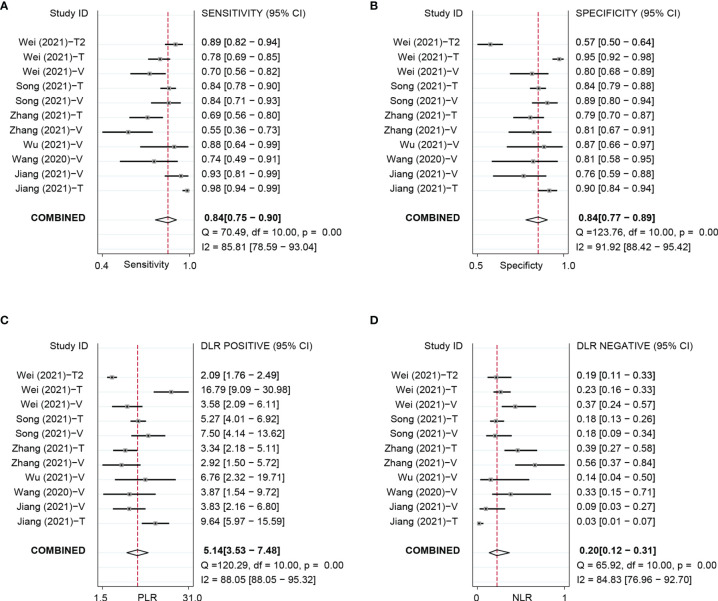
Forest plots based on DL model for preoperative prediction of MVI in HCC. DL, deep learning; MVI, microvascular invasion; HCC, hepatocellular carcinoma; DL, deep learning; MVI, microvascular invasion; HCC, hepatocellular carcinoma; T, training set; V, validationset; Wei (2021)-T1,model in training set based on MRI; Wei (2021)-T2, model in validation set based on CT.

**Figure 3:**
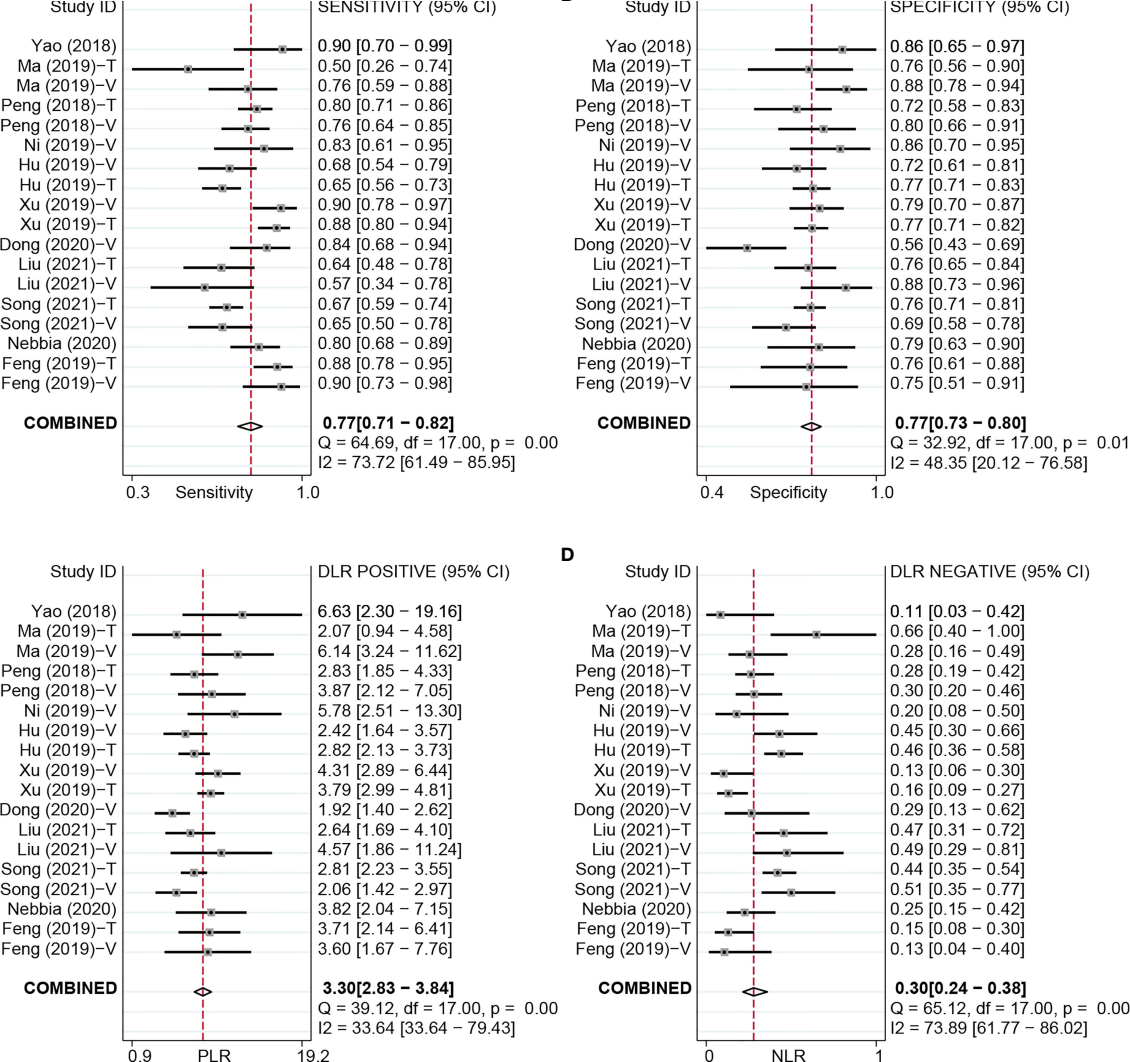
Forest plots based on NDL model for preoperative prediction of MVI in HCC. NDL, non-deep learning; MVI, microvascular invasion; HCC, hepatocellular carcinoma; T, training set; V, validation set.

**Figure 4:**
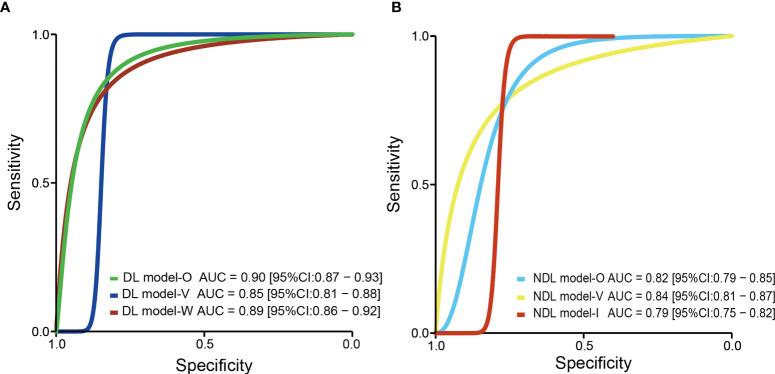
The pooled sROC curve of DL model **(A)** and NDL model **(B)**. sROC, summary receiver operating characteristic; DL, deep learning; NDL, non-deep learning.

Based on 11 DL models in all cohorts, there were 2,073 HCC patients, including 843 MVI-present and 1,230 MVI-absent. The diagnostic meta-analysis forest plots and the combined results are shown in **Figure 2**. Diagnostic threshold analysis showed that there was no significant threshold effect (Spearman’s correlation coefficient = −0.082 p = 0.811). The pooled sensitivity, specificity, PLR, and NLR of the DL model were 0.84 [95% CI: 0.75–0.90, I^2^ = 85.81%], 0.84 [95% CI: 0.77–0.89, I^2^ = 91.92%], 5.14 [95% CI: 3.53–7.48, I^2^ = 88.05%], and 0.2 [95% CI: 0.12–0.31, I^2^ = 84.83%], respectively. The AUC based on the summary ROC (sROC) curve was 0.90 [95% CI: 0.87–0.93; **Figure 4**]. The I^2^ values of sensitivity, specificity, PLR, and NLR indicated high heterogeneity. Influence analysis showed that the models of Jiang et al. and Wei et al. in their training sets could be the cause of the high heterogeneity. After the two models were excluded, I^2^ values markedly decreased (**Table 3**). Based on 9 DL models, there were 1,443 HCC patients, including 565 MVI-present and 878 MVI-absent. Analysis of diagnostic threshold showed that there was no significant threshold effect (Spearman’s correlation coefficient = −0.150 p = 0.700). The pooled sensitivity, specificity, PLR, and NLR of the DL model were 0.79 [95% CI: 0.71–0.85, I^2^ = 70.54%], 0.85 [95% CI: 0.80–0.89, I^2^ = 69.44%], 5.34 [95% CI: 3.79–7.52, I^2^ = 48.71%], and 0.25 [95% CI: 0.18–0.35, I^2^ = 74.00%], respectively. The AUC based on the sROC curve was 0.89 [95% CI: 0.86–0.92; **Figure 3**], which showed moderate diagnostic value. Studies in the DL group numbered less than ten, and thus meta-regression analysis could not be performed.

**Table 3:** Sensitivity, specificity, positive likelihood ratio, and negative likelihood ratio with subgroup analysis according to the number of tumors in NDL model group.

Analysis	No. of models	Pooled SE (95% CI)	I^2^ (%)	Pooled SP (95% CI)	I^2^ (%)	Pooled PLR (95% CI)	I^2^ (%)	Pooled NLR (95% CI)	I^2^ (%)	AUC
NDL model group	18	0.77 [0.71–0.82]	73.72	0.77 [0.73–0.80]	48.35	3.30 [2.83–3.84]	33.64	0.30 [0.24–0.38	73.90	0.82 [0.79–0.85]
NDL model in validation set	9	0.77 [0.70–0.83]	61.59	0.77 [[0.70–0.83]	72.85	3.42 [2.54–4.62]	53.76	0.29 [0.22–040]	63.21	0.84 [0.81–0.87]
DL model group	11	0.84 [0.75–0.90]	85.81	0.84 [0.77–0.89]	91.92	5.14 [3.53–7.48]	88.05	0.2 [0.12–0.31]	84.83	0.90 [0.87–0.93]
DL model in validation set	6	0.79 [0.56–0.86]	74.90	0.83 [0.78–0.87]	0.00	4.72 [3.46–6.44]	0.00	0.25 [0.15–0.42]	76.72	0.85 [0.81–0.88]
**Influence analysis in DL model group**										
Without Jiang-T	10	0.80 [0.73–0.86]	74.64	0.83 [0.75–0.88]	91.76	4.69 [3.24–6.78]	85.71	0.24 [0.17–0.33]	74.01	0.88 [0.85–0.91]
Without Wei-T2	10	0.83 [0.73–0.90]	85.95	0.86 [0.81–0.90]	68.70	5.88 [4.19–8.24]	56.24	0.20 [0.12–0.33]	85.23	0.91 [0.88–0.93]
Without both	9	0.79 [0.71–0.85]	70.54	0.85 [0.80–0.89]	69.44	5.34 [3.79–7.52]	48.71	0.25 [0.18–0.35]	74.00	0.89 [0.86–0.92]
**Subgroup analysis in NDL model group**										
Single tumor	8	0.69 [0.65–0.73]	43.26	0.77 [0.74–0.80]	32.54	2.98 [2.54–3.45]	0.00	0.41 [0.35–0.48]	39.30	0.79 [0.75–0.82]
Multiple tumor	10	0.84 [0.78–0.88]	0.00	0.78 [0.72–0.83]	60.09	3.67 [2.82–4.78]	35.97	0.17 [0.13–0.23]	0.00	0.88 [0.85–0.91]
**Subgroup analysis in NDL without ultrasound**	14	0.77 [0.71–0.83]	74.70	0.77 [0.75–0.80	13.48	3.42 [2.98–3.93]	6.36	0.29 [0.22–0.38]	76.24	0.79 [0.75–0.82]
Single tumor	8	0.70 [0.63–0.75]	52	0.78 [0.73–0.82]	44.46	3.10 [2.49–3.86]	4.84	0.39 [0.32–0.48]	51.80	0.81 [0.77–0.84]
Multiple tumor	6	0.87 [0.83–0.90]	0.00	0.78 [0.74–0.81]	0.00	3.93 [3.31–4.68]	0.00	0.17 [0.13–0.23]	0.00	0.90 [0.87–0.92]
**Subgroup analysis by AI algorithms**										
LASSO	8	0.75 [0.67–0.81]	72.72	0.76 [0.72–0.79]	10.70	3.05 [2.55–3.64]	0.00	0.34 [0.25–0.45]	70.09	0.77 [0.73–0.80]
SVM	6	0.81 [0.71–0.88]	72.65	0.81 [0.76–0.85]	3.48	4.14 [3.33–5.16]	0.00	0.24 [0.16–0.36]	77.04	0.85 [0.81–0.88]
CNN	6	0.82 [0.78–0.86]	57.42	0.84 [0.73–0.92]	95.38	5.28 [3.04–9.19]	91.72	0.21 [0.17–0.25]	40.47	0.87 [0.84–0.90]
3D-CNN	5	0.87 [0.67–0.96]	93.29	0.84 [0.78–0.88]	48.01	5.30 [3.44–8.16]	49.39	0.16 [0.05–0.46]	93.65	0.88 [0.85–0.90]
**Subgroup analysis by image**										
MRI	5	0.78 [0.67–0.87]	80.99	0.76 [0.70–0.81]	27.70	3.22 [2.48–4.19]	27.90	0.28 [0.18–0.45]	82.36	0.78 [0.74–0.81]
CT	9	0.76 [0.68–0.83]	72.36	0.80 [0.76, 0.83]	13.11	3.73 [3.12–4.45]	0.00	0.30 [0.22–0.41]	73.85	0.82 [0.78–0.85]

Jiang-T: DL model proposed by Jiang et al. in training set; Wei-T2: DL model based on CT proposed by Wei et al. in validation set; SE, sensitivity; SP, specificity; PLR, positive likelihood ratio; NLR, negative likelihood ratio; AUC, area under the curve; NDL, non-deep learning; DL, deep learning; AI, artificial intelligence; LASSO, least absolute shrinkage and selection operator; SVM, support vector machine; CNN, convolutional neural network.

### Non-Deep Learning Model for Preoperative Microvascular Invasion Evaluation

For the NDL model across all cohorts, there were 2,685 HCC patients, including 1,128 MVI-present and 1,557 MVI-absent. The diagnostic meta-analysis forest plots and combined results are shown in **Figure 3**. Diagnostic threshold analysis showed that there was no significant threshold effect (Spearman’s correlation coefficient = −0.089, p = 0.726). The pooled sensitivity, specificity, PLR, and NLR of the NDL model were 0.77 [95% CI: 0.71–0.82, I^2^ = 73.72%], 0.77 [95% CI: 0.73–0.80, I^2^ = 48.35%], 3.30 [95% CI: 2.83–3.84, I^2^ = 33.64%], and 0.30 [95% CI: 0.24–0.38, I^2^ = 73.89%], respectively. The AUC based on the sROC curve was 0.82 [95% CI: 0.79–0.85; **Figure 4**], which showed moderate diagnostic value. Heterogeneity between groups was considered moderate.

US is operator-dependent, and its imaging techniques are different from those of CT and MRI. To reduce the bias, studies (Hu, Yao, and Dong) using US were excluded, and a meta-analysis based on 14 NDL models using CT or MRI was performed. There were 2,059 HCC patients, consisting of 875 MVI-present and 1,184 MVI-absent. The diagnostic meta-analysis forest plots and combined results are shown in **Supplementary Figure S4** and **Table 3**. Diagnostic threshold analysis showed that there was no significant threshold effect (Spearman’s correlation coefficient = −0.089, p = 0.726). The pooled sensitivity, specificity, PLR, and NLR of the NDL model were 0.77 [95% CI: 0.71–0.83, I^2^ = 74.70%], 0.77 [95% CI: 0.75–0.80, I^2^ = 13.48%], 3.42 [95% CI: 2.98–3.93, I^2^ = 6.36%], and 0.29 [95% CI: 0.22–0.38, I^2^ = 76.24%], respectively. The AUC based on the sROC curve was 0.79 [95% CI: 0.75–0.82; **Figure 4**], which showed a moderate diagnostic value. Heterogeneity between groups was considered moderate. After studies using US were excluded, the I^2^ values of PLR were markedly decreased, but the I^2^ values of sensitivity, specificity, and NLR did not noticeably decrease.

### Deep Learning Model for Preoperative Microvascular Invasion Evaluation in Validation Set

Considering the effect of overfitting in the model training process, a meta-analysis based on DL models in the validation set was performed after removing the training set. Within the six included DL models in the validation set, there were 495 HCC patients, including 216 MVI-present and 279 MVI-absent. The diagnostic meta-analysis forest plots and combined results are shown in **Supplementary Figure S2**. Diagnostic threshold analysis showed that there was no significant threshold effect (Spearman’s correlation coefficient = 0.086, p = 0.872). The pooled sensitivity, specificity, PLR, and NLR of the DL model were 0.79 [95% CI: 0.67–0.88, I^2^ = 74.90%], 0.83 [95% CI: 0.78–0.87, I^2^ = 0.00%], 4.72 [95% CI: 3.46–6.44, I^2^ = 0.00%], and 0.25 [95% CI: 0.15–0.42, I^2^ = 76.72%], respectively. The AUC based on the sROC curve was 0.85 [95% CI: 0.81–0.88; **Figure 4**], which showed moderate diagnostic value. After the removal of the training set, the I^2^ values were markedly decreased, while heterogeneity between included models was still considered notable in terms of NLR. There was no significant difference in all effect sizes between the models in all cohorts and models in the validation set.

### Non-Deep Learning Model for Preoperative Microvascular Invasion Evaluation in Validation Set

Considering the effect of overfitting in the model training process, a meta-analysis based on an NDL model in the validation set was performed. Of the nine included NDL models in the validation set, there were 926 HCC patients, composing 381 MVI-present and 545 MVI-absent. The diagnostic meta-analysis forest plots and combined results are shown in **Supplementary Figure S3**. Diagnostic threshold analysis showed that there was no significant threshold effect (Spearman’s correlation coefficient = 0.192, p = 0.620). The pooled sensitivity, specificity, PLR, and NLR of the NDL model were 0.77 [95% CI: 0.70–0.83, I^2^ = 61.59%], 0.77 [95% CI: 0.70–0.83, I^2^ = 72.85%], 3.42 [95% CI: 2.54–4.62, I^2^ = 53.76%], and 0.29 [95% CI: 0.22–0.40, I^2^ = 63.21%], respectively. The AUC based on the sROC curve was 0.84 [95% CI: 0.81–0.87], which showed moderate diagnostic value. After the removal of the training set, heterogeneity between groups was considered moderate. There was no significant difference in all effect sizes between the models from all cohorts and models in the validation set.

### Meta-Regression Analysis and Subgroup Analysis

We observed substantial heterogeneity in the performance of the NDL group, with I^2^ being 73.72%, 48.35%, 33.64%, and 73.89% for the pooled sensitivity, specificity, PLR, and NLR, respectively. As US may result in a noticeable bias, we excluded studies using US and then performed the meta-regression analysis. The results of meta-regression analysis are presented in **Tables S4**, **S5**. The results showed that in the univariate meta-regression model, 10 covariates were significantly associated with study heterogeneity. Therefore, we believe that these variates may influence prediction accuracy in the NDL group. In the multivariate meta-regression model, the number of tumors was strongly associated with study heterogeneity.

We conducted an additional subgroup analysis based on the number of tumors (**Table 3**). In it, I^2^ values of the two subgroups were markedly decreased. The I^2^ of the single tumor subgroup was 43.26%, 0%, and 39.28% for the pooled sensitivity, PLR, and NLR, respectively. The I^2^ of the multiple tumor subgroup was 0% and 0% for the pooled sensitivity and NLR, respectively. Except for the pooled specificity and PLR, significant differences between the two subgroups were observed in the pooled sensitivity, AUC, and NLR. The results of subgroup analysis using the AI algorithm (LASSO and SVM) and image (CT and MRI) are shown in **Table 3**. There was no significant difference between the image and AI algorithms in the NDL group. For AI algorithms in the NDL group, SVM is significantly superior to LASSO for the pooled AUC (0.77 [0.73–0.80] *vs.* 0.85 [0.81–0.88]). There was no significant difference between CNN and 3D-CNN. Generally, DL models (3D-CNN and CNN) are significantly superior to LASSO, and there was no significant difference between DLAs and SVM.

### Testing for Publication Bias

Deeks’ funnel plot asymmetry test showed no significant publication bias with p-values of 0.42 and 0.22 for the DL group and NDL group, respectively.

## Discussion

### Performance of Deep Learning and Non-Deep Learning Models

In this study, NDL models and DL models were compared. The NDL models had a moderate diagnostic value for MVI prediction in HCC, with pooled sensitivity, specificity, PLR, NLR, and AUC values of 0.77, 0.77, 3.30, 0.30, and 0.82, respectively. The DL models, including the CNN model and 3D-CNN model, had moderate diagnostic values that were similar to those of the NDL models, with pooled sensitivity, specificity, PLR, NLR, and AUC values of 0.84, 0.84, 5.14, 0.2, and 0.90, respectively. All these effect sizes showed that models using the DL method had a higher performance for preoperative prediction of MVI in HCC and had a statistically significant difference in diagnostic value in terms of AUC. When comparing DL models with NDL models in the validation set, there was no significant difference in any of these factors. A reasonable interpretation is that the sample sizes of the DL model group were too small, and the heterogeneity in both the NDL and DL model groups was notable. However, there is reliable evidence to support that the model using the DL method may have a higher performance and be more suitable for preoperative MVI prediction.

By analyzing radiomics features from images, building a prediction model using NDL methods had been widely applied in MVI prediction (44–48) and prediction domain of other cancers (13–16). NDL models based on radiomics features had been proved to be better than a model based on radiological characteristics or clinical characteristics (44, 45). For the NDL models included in this study, analyzing radiomics features assisted by NDLAs is an advanced technique for MVI prediction, but one of the shortcomings of radiomics is that the method is based on handcrafted feature extractors, which require extensive work and manpower. In addition, the main limitation is that radiomics features are human-designed and dependent on domain-specific expertise.

A DL method, CNN, was proven powerful in medical imaging (49), with superior performance as compared to NDL based on radiomics features. The advantage of DL is that feature extraction in the learning process is not required, avoiding defects in human-designed features in radiomics analysis. Since classifier training, feature selection, and classification of DL occur simultaneously, we needed only input images, rather than clinical data, radiological features, or radiomics features. Feature selection and classification of DL occur simultaneously during classifier training. The main power of a CNN lies in a CNN architecture consisting of a series of layers of convolution filters, akin to low-level vision processing in the human brain, which allows for the extraction of a set of discriminating features at multiple levels of abstraction. However, training a deep CNN is challenging. The main difficulties are that CNNs require a large amount of labeled training data and large computational and memory requirements and that training a deep CNN is often complicated by overfitting and convergence issues and the lack of interpretability. Jiang et al. provided a new means to partly explain how DL can identify MVI status.

The main difference in 3D-CNNs is that the input data are three-dimensional image data. In the included studies, Wu et al. proposed a 3D-CNN model with a DSN based on pre-contrast, APs, and PVPs in MR images with an AUC value of 0.9255. A 3D-CNN model proposed by Song et al. with DSN based on eight MRI sequences obtained the highest AUC value of 0.915 in the testing cohort. Another 3D-CNN model proposed by Jiang et al. based on AP, PVP, and DP CT sequences in the validation set achieved 0.906 [95% CI: 0.821–0.960]. In the studies by Song et al. and Jiang et al., the two 3D-CNN models performed excellently in MVI prediction.

### The Value of Artificial Intelligence Algorithms for Microvascular Invasion Prediction

For AI algorithms, we performed a subgroup analysis, and results showed that DL is generally superior to NDL and that in NDL, SVM is significantly superior to LASSO. The advantage of DL has been previously discussed. The reason for the better performance of SVM than LASSO may be that the combination of modeling by SVM, and feature selection by LASSO has an advantage over than LASSO regression model only using LASSO for feature selection. SVM is a good classifier, but it may not get good performance when it is directly used for classification, but if it can be combined with a good feature selection algorithm, the classification performance will be greatly improved.

### The Potential Clinical Value of Convolutional Neural Network Models

A CNN model proposed by Wei et al. based on T2W1, T1WI, AP, PVP, and HBP MRI sequences achieved an AUC value of 0.802 in an independent external validation cohort. Furthermore, in the study by Song et al., survival analysis demonstrated that patients with DLC-predicted MVI status were associated with poor overall survival and recurrence-free survival, whereas in a study by Jiang et al., based on the MVI status predicted by the 3D-CNN model, the mean recurrence-free survival was significantly better in the predicted MVI-negative group than in the predicted MVI-positive group [64.06 *vs.* 31.05 months, p = 0.027]. In the study by Wei et al., survival analysis indicated that CNN models could stratify groups with high and low risks in terms of progression-free survival and overall survival (p < 0.05). These key findings indicate that the DL model can provide a non-invasive approach to accurately evaluate MVI, with the potential to facilitate clinical decision-making and assess patient prognosis.

### Prediction Values of Various Types of Input Data

CT or MRI data from arterial and portal phases were used to build the prediction model and proved powerful for MVI prediction in 13 of the included studies. Jiang et al. proposed a 3D-CNN model based on AP, PVP, and DP of CT images, which achieved an AUC value of 0.906. For five of the included studies, the AUC value of the prediction model based on AP and PVP of MR images ranged from 0.80 to 0.94. Five of the included studies in the DL group used MR images, and three studies in NDL used MR images. Among them, Wu et al. proposed a 3D-CNN model with DSN based on pre-contrast, AP, and PVP phases in MR images with an AUC value of 0.925. A meta-analysis of MRI features for predicting MVI of HCC performed by Hong et al. showed a similar conclusion that arterial enhancement and arterial peritumoral enhancement were significant predictors for MVI of HCC (50). However, in this study, the results of meta-regression showed no significant difference in the AP or PVP. The probable reasons for this were high heterogeneity and that the number of relevant original studies was small. Diffusion-weighted imaging (DWI) is an MRI sequence that can reflect the motion state of water molecules *in vivo* (51). Nebbia et al. built an SVM model based on a DWI sequence and performed worse than the AP or PVP sequence. However, in the study by Song et al., a CNN model based on eight MRI sequences, including DWI, AP, and PVP, achieved an AUC value of 0.915. Features from the DWI sequence, as complementary to AP and PVP, could further improve the performance of MVI prediction. Wang et al. suggested that deep features derived from higher b values yield better performance for MVI prediction, implying that DWI with a higher b value might be better for MVI prediction. Chen et al. indicated that the ADC value can also be used to evaluate MVI and has a diagnostic efficacy similar to the 20-min T1 relaxation time [AUC, 0.850 *vs.* 0.846]. Wu et al. indicated that due to the overflow of contrast agents from the tumor region in the delayed phase, and the tissue cellularity and vascularity within the tumor becoming unclear, information from the delayed phase sequence has worse predictive performance and may not fit MVI prediction. US was mainly used for MVI prediction in NDL models, and the results showed that the AUC value of models based on US ranged from 0.726 to 0.731, lower than that based on CT and MRI (35, 37, 40).

The HBP of contrast-enhanced liver MRI with gadoxetate disodium (Gd-EOB-DTPA) has the value of significantly increasing sensitivity and specificity in liver diagnosis (51, 52) and predicting MVI in HCC (33). Hong et al. performed a meta-analysis based on MRI features for MVI prediction, with the results showing that peritumoral hypointensity on HBP was the MRI feature most suggestive of MVI with the pooled diagnostic odds ratio (DOR) and pooled positive LR being 8.2 and 5.0, respectively (50). Chen et al. built an SVM model based on the hepatobiliary phase sequence of Gd-EOB-DTPA MRI, with a performance of 0.942 AUC value, higher than the AP and PVP sequences for MVI prediction. In this study, since the results showed that there was no significant difference between MRI and CT, analysis based on MRI features for MVI prediction did not yield significant results.

Within the DL group, the models proposed by Wang et al. and Zhang et al. obtained lower performance with AUC values of 0.79 and 0.72, respectively. The possible reasons are the differences in the types of input data. Notably, the input data of the two DL models did not include the imaging data in AP and PVP. However, further studies are needed to confirm this hypothesis.

### CT *vs.* MRI in Artificial Intelligence Algorithms for Microvascular Invasion Prediction

Compared with CT, MRI can better describe the characteristics of soft tissue, atomic signal intensity, and lesion enhancement, as well as provide more information on tissue function.

For models using 3D-CNN algorithms in the DL group, two studies used MRI techniques (Wu and Zhang), and one study used a CT imaging technique (Jiang). We observed that the training set containing 3D-CNN models using CT by Jiang et al. achieved the highest AUC value of 0.98. In the validation set, Wu et al. proposed 3D-CNN models using MRI, which had the highest AUC value of 0.926. Since the number of studies was too small, a meta-analysis could not be performed. For models using CNN algorithms in the DL group, two studies used MRI (Song and Wang), and one study used CT and MRI (Wei). Wei et al. built DL models for preoperative prediction of MVI based on CT and MR images. The results of the meta-analysis showed superior predictive power from MRI compared to CT (AUC: 0.812 *vs.* 0.736, p = 0.039).

In this study, meta-regression analysis was performed for models in the NDL group. The results showed that imaging techniques may be influencing factors of prediction power in the NDL group but not independently influencing factors. There was no significant consequence of the predictive power of MRI being superior to CT (AUC: 0.78 [0.74–0.81] *vs.* 0.82 [0.78–0.85]).

Overall, our results showed that, in the DL model group, especially the CNN model, MRI was superior to CT in the prediction of MVI. However, there was no significant advantage that MRI had in MVI prediction, compared with CT. Recently, Meng et al. compared the performance of radiomics models based on CT and MRI for MVI prediction (53). The results showed that CT and MRI had a comparable performance for MVI prediction in a single HCC. Studies comparing the performance of AI algorithms based on CT and MRI for MVI prediction are too small and can be excluded.

### Deep Learning Models Combined With Clinical Characteristics

Previous studies have predicted MVI using clinical characteristics, such as tumor number and size, alpha fetoprotein (AFP), protein induced by vitamin K absence or antagonist (PIVKAII), and serum component index. The AUC of these predictors varies from 0.529 to 0.81 (18–23). In this study, some clinical variables [tumor size, AFP, tumor margin, internal arteries, and International normalized ratio (INR)] that were recognized as predictive values were selected by statistical analysis and then integrated with the DL model to further improve predictive performance. Clinical variables recognized as MVI-prediction values were tumor size in 11 studies and AFP in nine studies; others are shown in **Table S3**. Some studies using radiomics combined with clinical parameters achieved better outcomes, ranging from 0.796 to 0.899 for AUC (36, 41, 54).

### Number of Tumors as One Source of Heterogeneity

In addition, we performed a subgroup analysis according to the number of tumors, and the results showed that the number of tumors was one of the sources of heterogeneity. Models based on HCC patients with multiple tumors performed better with the pooled AUC value of 0.88 [0.85–0.91] and sensitivity of 0.84 [0.78–0.88] than single tumors with 0.79 [0.75–0.82] and 0.69 [0.65–0.73], respectively. In HCC patients, having multiple tumors was regarded as a variable that had strong associations with a high risk of MVI. This could cause these models to more easily identify the MVI status in HCC patients with multiple tumors than single tumors. However, because the number of models in the meta-analysis was relatively small, the results of the subgroup analysis need to be interpreted with caution.

### Trends, Challenges, and Suggestions

According to the analysis of the existing MVI prediction models presented above, the diagnostic accuracy of CNNs for preoperative MVI prediction has achieved spectacular progress in terms of sensitivity, specificity, PLR, NLR, and AUC. However, there is much room for improvement due to existing challenges, as well as many options for future research.

### Methodological Trends

In six studies using DL in this meta-analysis, CNNs have been the main methods for MVI prediction. The six studies used ensemble learners of CNNs, which is an approach for integrating multiple learner branches into a single fusion model to improve the prediction of MVI in HCC (55). In each learner branch, fully convolutional networks and softmax layers were employed to calculate the predicted results. In the studies by Wang et al. and Wu et al., a DSN that combines the loss functions of each CNN learner branch was designed for the proposed DL network. Jiang et al. and Song et al. designed specific architectures as CNN branches for feature extraction, with their final DL models achieving AUCs of 0.906 and 0.915, respectively.

### Challenges and Suggestions

#### Lack of Datasets With Large Numbers of Cases

One of the critical barriers in the application of DL for MVI prediction based on medical imaging data is the lack of datasets with large numbers of samples. It is noted that the process of training DL models using CNNs requires a huge amount of data. However, their collection is still very difficult in clinical practice.

To mitigate this problem, new techniques for generating synthetic medical images could be developed. For instance, Zhang et al. generated an augmented training set by randomly rotating the original imaging dataset at a full 360° angle. Moreover, Wang et al. used an image resampling method to generate more samples for training a DL network.

#### Generalizability

Typically, a specific model that performs very well on a specific task may not be generalized to other tasks. Heterogeneity could be one of the major reasons why a specific model cannot be generalized to other tasks. The sources of heterogeneity are various imaging modalities, and different medical scanners operate under different settings and datasets. This issue could also be alleviated by developing methods that can be validated on images of different types. In addition, research on the effect of scanner settings (reconstruction techniques, parameters, etc.) on MVI prediction is expected.

#### Lack of Interpretability

The black box problem has been one of the major criticisms of the deep CNN approach, implying that the system struggles to provide evidence to support clinical decisions. Better interpretability would contribute to understanding how the MVI status is generated. This may lead to more accurate and reliable clinical decisions.

To improve the accuracy of diagnosis and interpretability of DL models, new approaches for both radiomics and semantic feature analysis in screening data can be developed. For example, to improve the interpretability of the 3D-CNN model, Jiang et al. attempted to predict the 15 most important variables selected by the XGBoost method, and the results indicated that the CNN model could predict the status of MVI partly based on the explainable features utilized in clinical practice.

#### Potential Value of Clinical Application

Several studies (Song et al., Jiang et al., and Wei et al.) performed survival analysis that showed that the patients with CNN-predicted MVI status were associated with poor survival after resection, suggesting the strong clinical value of the CNN model in preoperatively identifying HCC patients with poor prognosis and guiding the resection range. However, there is no evidence from prospective studies or clinical trials. Thus, in the future, some prospective research and clinical trials concerning CNN models for MVI prediction that guide clinical decisions are expected.

### Contributions and Limitations

Our meta-analysis of DL methods and NDL methods for preoperative MVI prediction in HCC patients has several advantages. First, this study involving 16 studies and 4,759 HCC cases is the first systematic review and meta-analysis of preoperative MVI prediction in HCC patients by comparing DL and NDL methods. Second, DL models perform better than NDL models in terms of the accuracy of MVI prediction, methodology, and cost-effectiveness.

This study has some limitations. First, all included studies were retrospective, inevitably causing a patient selection bias. Second, this study only included six studies for DL methods in MVI prediction because CNNs are powerful tools for a broad range of computer vision tasks applied in medical imaging in recent years, and training a CNN requires a large sample size, which is difficult in clinical tasks. Third, only one included study used an independent external validation cohort to assess the performance of DL models. Finally, study heterogeneity was significant across the included studies.

## Conclusions

This meta-analysis demonstrates the high diagnostic accuracy of NDL and DL methods for the prediction of MVI and their promising potential for application in clinical decision-making. Multicentral validation and larger sample sizes are required for more definitive conclusions. DL models perform better than NDL models in terms of the accuracy of MVI prediction, methodology, and cost-effectiveness. CT or MRI data from the arterial and portal phases were used to build a prediction model and were proved effective for MVI prediction. Clinical variables, such as tumor size and AFP, were recognized as MVI prediction values. Studies of DL models for MVI prediction for HCC patients with single tumors are expected.

## Data Availability Statement

The original contributions presented in the study are included in the article/**Supplementary Material**. Further inquiries can be directed to the corresponding author.

## Author Contributions

JZ designed the study drafted the manuscript. SH and JZ were responsible for the collection and analysis of the research information. JZ, SH, YX, and JW critically and carefully revised this manuscript. The authors read and approved the final manuscript.

## Conflict of Interest

The authors declare that the research was conducted in the absence of any commercial or financial relationships that could be construed as a potential conflict of interest.

## Publisher’s Note

All claims expressed in this article are solely those of the authors and do not necessarily represent those of their affiliated organizations, or those of the publisher, the editors and the reviewers. Any product that may be evaluated in this article, or claim that may be made by its manufacturer, is not guaranteed or endorsed by the publisher.

## Supplementary Material

Supplementary Figure S1 Quality assessment of the included studies based on the QUADAS-2 scale.

Supplementary Figure S2 Forest plots based on deep learning model in validation set for preoperative prediction of MVI in HCC.

Supplementary Figure S3 Forest plots based on non-deep learning model in validation set for preoperative prediction of MVI in HCC.

Supplementary Figure S4 Forest plots based on non-deep learning model excluding studies using ultrasound for preoperative prediction of MVI in HCC.

## Abbreviations

HCC, hepatocellular carcinoma; MVI, microvascular invasion; DLAs, deep learning algorithms; NDLAs, non-deep learning algorithms; US, ultrasound; PLR, positive likelihood ratio; NLR, negative likelihood ratio; DL, deep learning; NDL, non-deep learning; DLC, the deep learning model with clinical variables; DSN, deep supervision network; AFP, alpha fetoprotein; PIVKAII, vitamin K absence or antagonist; AP, arterial phase; PVP, portal venous phase; SVM, support vector machine; XGBoost, extreme gradient boosting; LR, logistic regression; LASSO, least absolute shrinkage and selection operator; CNN, convolutional neural network; HBP, delayed hepatobiliary phase.
