# Supplementary figures and images for: Diagnostic Accuracy of Artificial Intelligence Based on Imaging Data for Preoperative Prediction of Microvascular Invasion in Hepatocellular Carcinoma: A Systematic Review and Meta-Analysis
Source: Front Oncol. 2022 Feb 24;12:763842. doi: 10.3389/fonc.2022.763842 (PMC8907853; doi:10.3389/fonc.2022.763842)

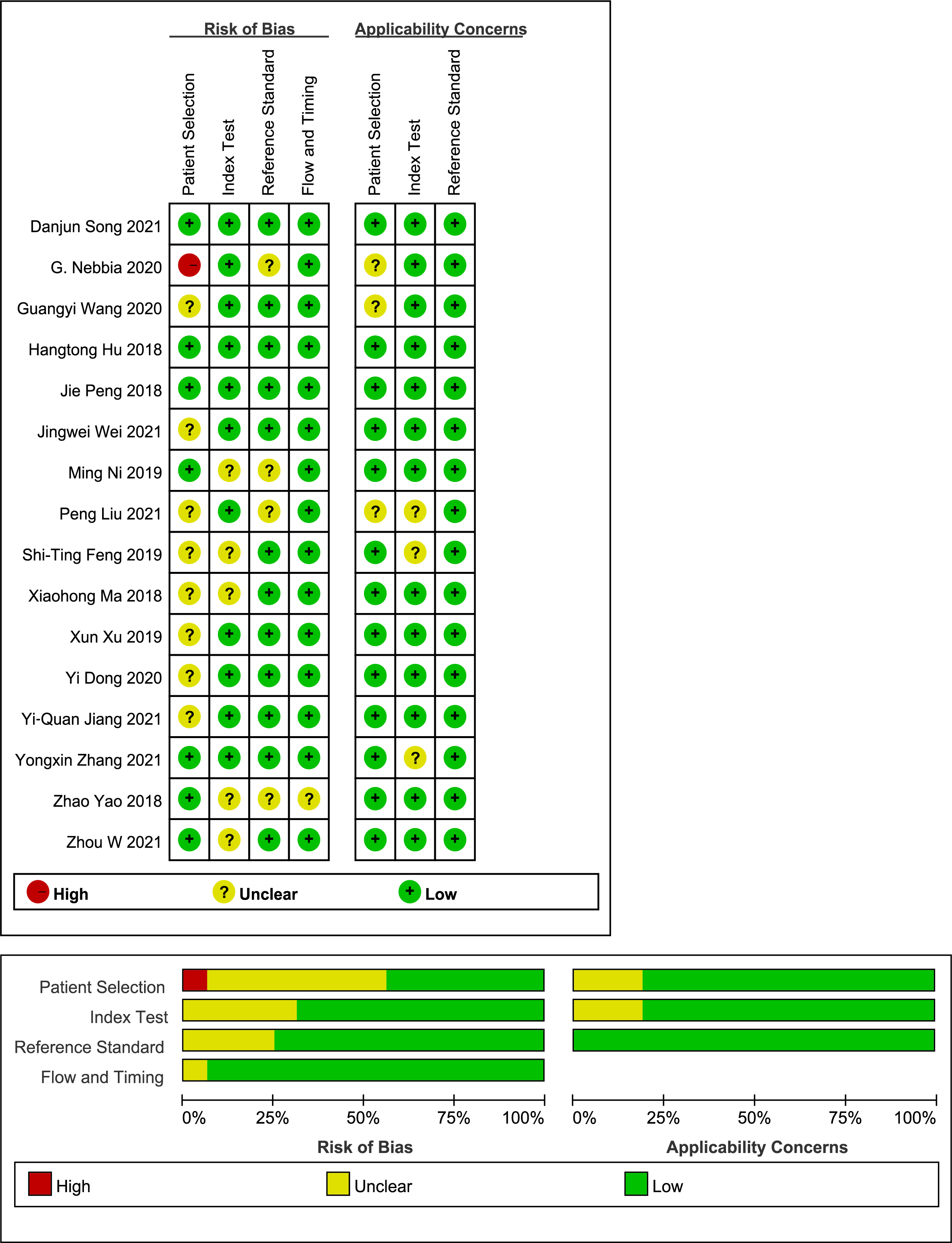

Supplement: Supplementary Figure S1 — Quality assessment of the included studies based on the QUADAS-2 scale. [file DataSheet_2.zip › Supplementary Figure 1.tif]

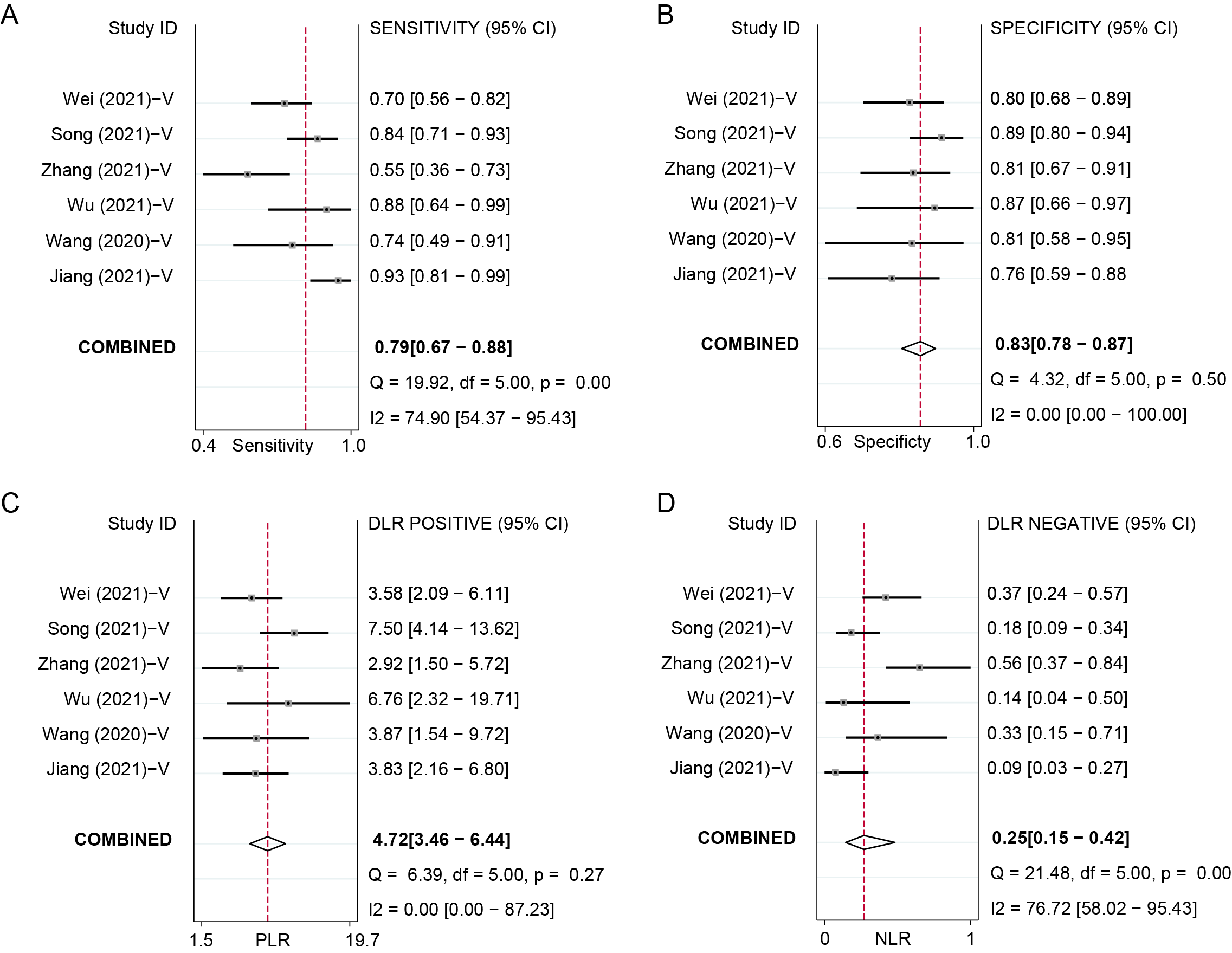

Supplement: Supplementary Figure S1 — Quality assessment of the included studies based on the QUADAS-2 scale. [file DataSheet_2.zip › Supplementary Figure 2.TIF]

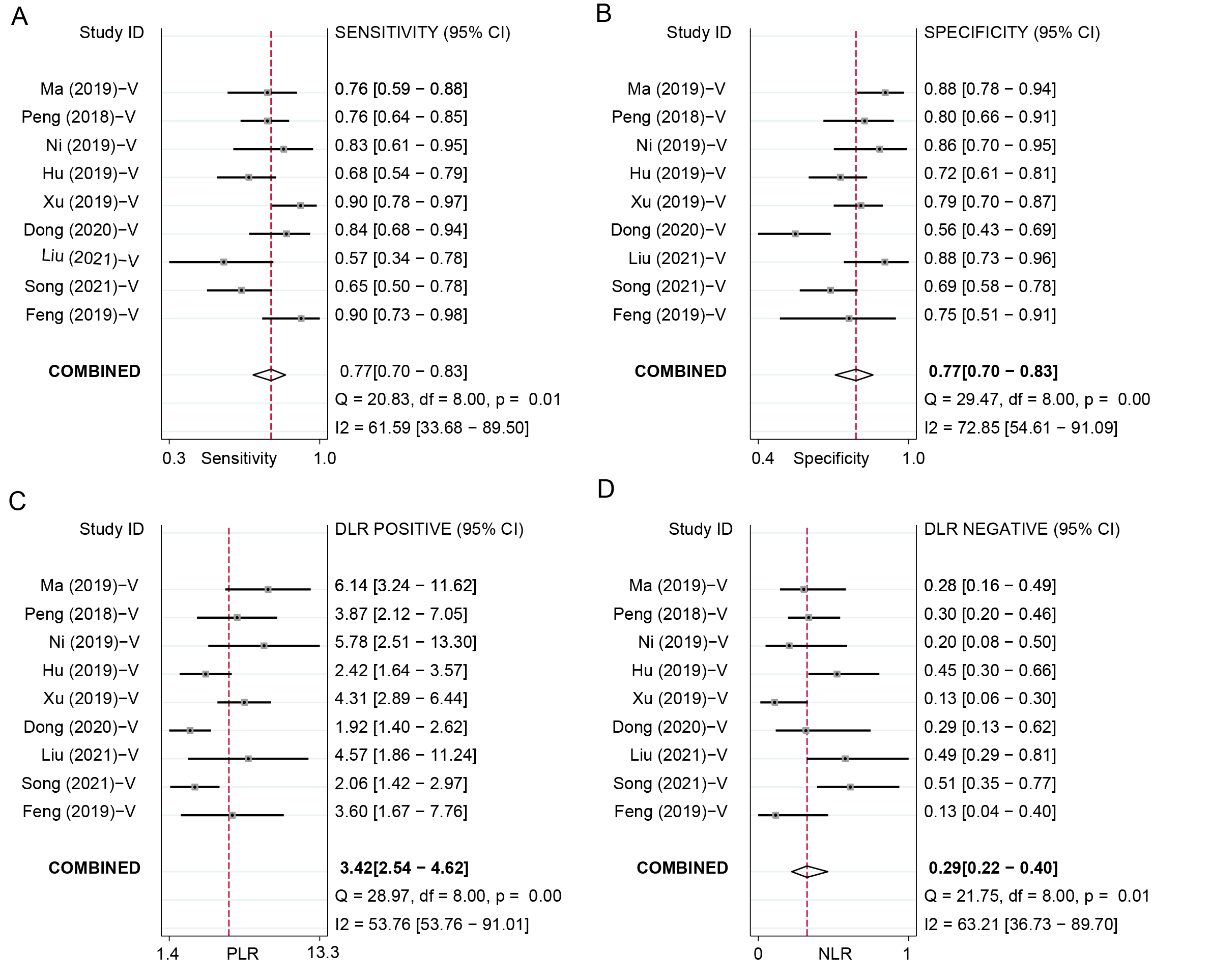

Supplement: Supplementary Figure S1 — Quality assessment of the included studies based on the QUADAS-2 scale. [file DataSheet_2.zip › Supplementary Figure 3.tif]

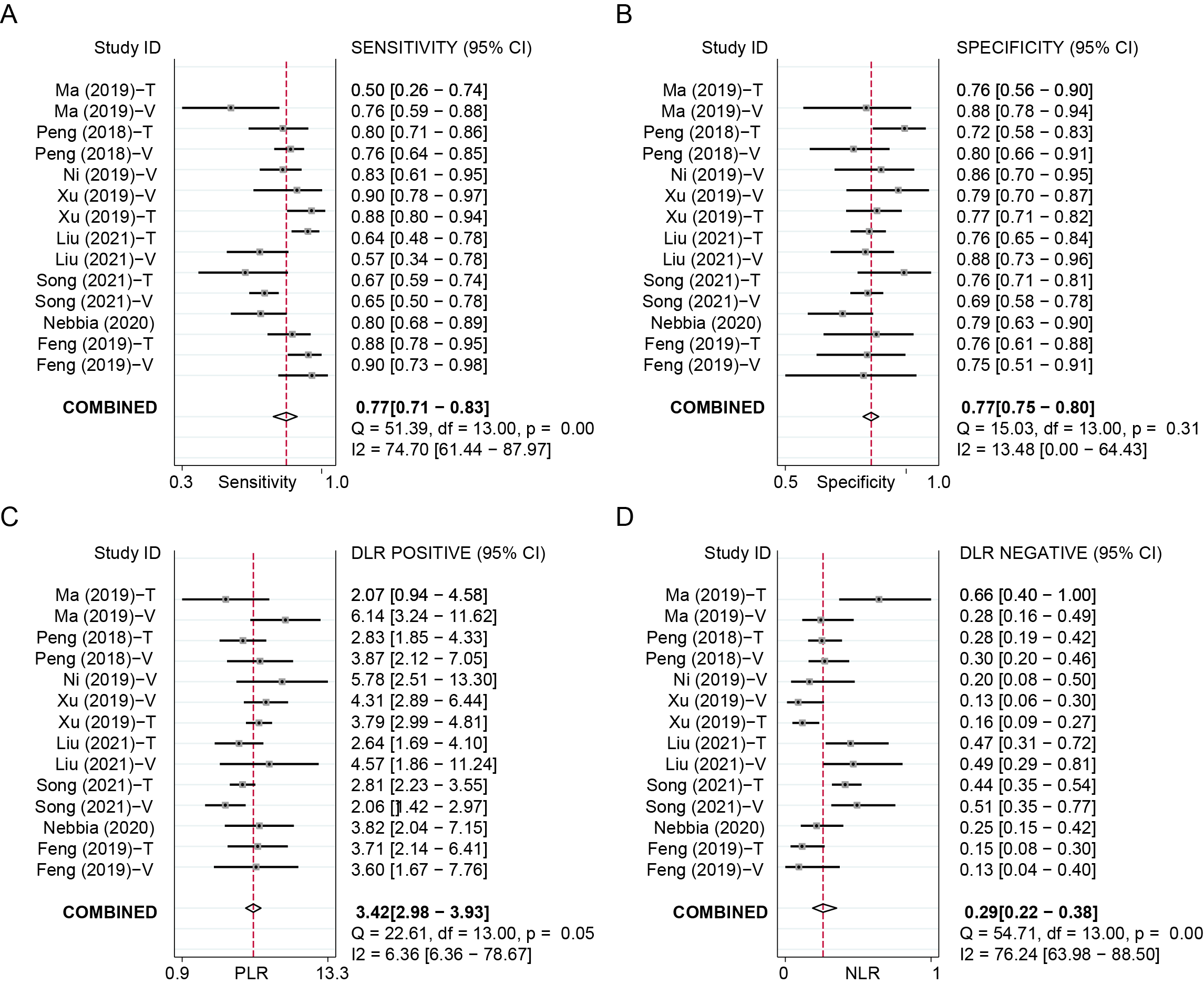

Supplement: Supplementary Figure S1 — Quality assessment of the included studies based on the QUADAS-2 scale. [file DataSheet_2.zip › Supplementary Figure 4.tif]
