# Supplementary material for: Diagnostic Accuracy of Artificial Intelligence Based on Imaging Data for Preoperative Prediction of Microvascular Invasion in Hepatocellular Carcinoma: A Systematic Review and Meta-Analysis
Source: Front Oncol. 2022 Feb 24;12:763842. doi: 10.3389/fonc.2022.763842 (PMC8907853; doi:10.3389/fonc.2022.763842)
Supplement: Supplementary file 2 [file DataSheet_1.docx]

**Search Strategy**

Papers describing the use of AI or machine learning or deep learning for the predication of hepatocellular carcinoma were reviewed. We searched the following databases: PubMed and Web of Science. All the English publications until June 14, 2021 will be searched without any restriction of countries or article type.

| **Mesh** | **Liver Neoplasms** | **Artificial Intelligence,**  **deep learning,**  **machine learning** | **diagnosis** |
| --- | --- | --- | --- |
| **Free words** | Liver Neoplasm OR Hepatic Neoplasms OR Hepatic Neoplasm OR Cancer of Liver OR Hepatocellular Cancer OR Hepatocellular Cancers OR Hepatic Cancer Hepatic Cancers OR Liver Cancer OR Liver Cancers OR Cancer of the Liver OR hepatocellular carcinoma OR Hepatocellular Carcinomas OR Liver Cell Carcinoma OR Liver Cell Carcinomas OR Hepatoma OR Hepatomas | Artificial Intelligence OR Computational Intelligence OR Machine Intelligence OR Computer Reasoning OR AI OR Computer Vision System OR Knowledge Acquisition OR Knowledge Representation OR Machine Learning OR Deep Learning OR Hierarchical Learning OR Transfer Learning or Computer aided | Search terms come from Health Information Research Unit - HIRU ~ Search Strategies for MEDLINE in Ovid Syntax and the PubMed translation (mcmaster.ca) |

**Search Strategy**

**Search terms in PubMed：**

((("Liver Neoplasms"[Mesh]) OR (((((((((((((((((((((Neoplasms, Hepatic[Title/Abstract]) OR (Neoplasm, Liver[Title/Abstract])) OR (Liver Neoplasm[Title/Abstract])) OR (Neoplasms, Liver[Title/Abstract])) OR (Hepatic Neoplasms[Title/Abstract])) OR (Hepatic Neoplasm[Title/Abstract])) OR (Neoplasm, Hepatic[Title/Abstract])) OR (Cancer of Liver[Title/Abstract])) OR (Hepatocellular Cancer[Title/Abstract])) OR (Cancers, Hepatocellular[Title/Abstract])) OR (Hepatocellular Cancers[Title/Abstract])) OR (Hepatic Cancer[Title/Abstract])) OR (Cancer, Hepatic[Title/Abstract])) OR (Cancers, Hepatic[Title/Abstract])) OR (Hepatic Cancers[Title/Abstract])) OR (Liver Cancer[Title/Abstract])) OR (Cancer, Liver[Title/Abstract])) OR (Cancers, Liver[Title/Abstract])) OR (Liver Cancers[Title/Abstract])) OR (Cancer of the Liver[Title/Abstract])) OR (Cancer, Hepatocellular[Title/Abstract]))) AND (((("Artificial Intelligence"[Mesh]) OR ("Deep Learning"[Mesh])) OR ("Machine Learning"[Mesh])) OR (((((((((((((((((((((((((Intelligence, Artificial[Title/Abstract]) OR (Computational Intelligence[Title/Abstract])) OR (Intelligence, Computational[Title/Abstract])) OR (Machine Intelligence[Title/Abstract])) OR (Intelligence, Machine[Title/Abstract])) OR (Computer Reasoning[Title/Abstract])) OR (Reasoning, Computer[Title/Abstract])) OR (AI (Artificial Intelligence)[Title/Abstract])) OR (Computer Vision Systems[Title/Abstract])) OR (Computer Vision System[Title/Abstract])) OR (System, Computer Vision[Title/Abstract])) OR (Systems, Computer Vision[Title/Abstract])) OR (Vision System, Computer[Title/Abstract])) OR (Vision Systems, Computer[Title/Abstract])) OR (Knowledge Acquisition (Computer)[Title/Abstract])) OR (Acquisition, Knowledge (Computer)[Title/Abstract])) OR (Knowledge Representation (Computer)[Title/Abstract])) OR (Knowledge Representations (Computer)[Title/Abstract])) OR (Representation, Knowledge (Computer)[Title/Abstract])) OR (Learning, Machine[Title/Abstract])) OR (Transfer Learning[Title/Abstract])) OR (Learning, Transfer[Title/Abstract])) OR (Learning, Deep[Title/Abstract])) OR (Hierarchical Learning[Title/Abstract])) OR (Learning, Hierarchical[Title/Abstract])))) AND (sensitiv*[Title/Abstract] OR sensitivity and specificity[MeSH Terms] OR (predictive[Title/Abstract] AND value*[Title/Abstract]) OR predictive value of tests[MeSH Term] OR accuracy*[Title/Abstract])

**Search terms Web of science：#1 AND #2 AND #3**

#1

TS=(Liver Neoplasms or Neoplasms, Hepatic or Neoplasms, Liver or Liver Neoplasm or Neoplasm, Liver or Hepatic Neoplasms or Hepatic Neoplasm or Neoplasm, Hepatic or Cancer of Liver or Hepatocellular Cancer or Cancers, Hepatocellular or Hepatocellular Cancers or Hepatic Cancer or Cancer, Hepatic or Cancers, Hepatic or Hepatic Cancers or Liver Cancer or Cancer, Liver or Cancers, Liver or Liver Cancers or Cancer of the Liver or Cancer, Hepatocellular)

#2

TS=(machine learning or deep learning or Artificial Intelligence or Intelligence, Artificial or Computational Intelligence or Intelligence, Computational or Machine Intelligence or Intelligence, Machine or Computer Reasoning or Reasoning, Computer or AI (Artificial Intelligence) or Computer Vision Systems or Computer Vision System or System, Computer Vision or Systems, Computer Vision or Vision System, Computer or Vision Systems, Computer or Knowledge Acquisition (Computer) or Acquisition, Knowledge (Computer) or Knowledge Representation (Computer) or Knowledge Representations (Computer) or Representation, Knowledge (Computer) or Learning, Machine or Transfer Learning or Learning, Transfer or Learning, Deep or Hierarchical Learning or Learning, Hierarchical)

#3

TS=(sensitiv* or sensitivity and specificity or predictive or value* or predictive value of tests or accuracy* )

**Search terms in Embase**：**#1 AND #2 AND #3**

#1

('neoplasms, hepatic':ab,ti OR 'neoplasms, liver':ab,ti OR 'liver neoplasm':ab,ti OR 'neoplasm, liver':ab,ti OR 'hepatic neoplasms':ab,ti OR 'hepatic neoplasm':ab,ti OR 'neoplasm, hepatic':ab,ti OR 'cancer of liver':ab,ti OR 'hepatocellular cancer':ab,ti OR 'cancers, hepatocellular':ab,ti OR 'hepatocellular cancers':ab,ti OR 'hepatic cancer':ab,ti OR 'cancer, hepatic':ab,ti OR 'cancers, hepatic':ab,ti OR 'hepatic cancers':ab,ti OR 'liver cancer':ab,ti OR 'cancer, liver':ab,ti OR 'cancers, liver':ab,ti OR 'liver cancers':ab,ti OR 'cancer of the liver':ab,ti OR 'cancer, hepatocellular':ab,ti) OR (('liver'/exp OR liver) AND ('neoplasms'/exp OR neoplasms))

#2

'intelligence, artificial':ab,ti OR 'computational intelligence':ab,ti OR 'intelligence, computational':ab,ti OR 'machine intelligence':ab,ti OR 'intelligence, machine':ab,ti OR 'computer reasoning':ab,ti OR 'reasoning, computer':ab,ti OR 'ai (artificial intelligence)':ab,ti OR 'computer vision systems':ab,ti OR 'computer vision system':ab,ti OR 'system, computer vision':ab,ti OR 'systems, computer vision':ab,ti OR 'vision system, computer':ab,ti OR 'vision systems, computer':ab,ti OR 'knowledge acquisition (computer)':ab,ti OR 'acquisition, knowledge (computer)':ab,ti OR 'knowledge representation (computer)':ab,ti OR 'knowledge representations (computer)':ab,ti OR 'representation, knowledge (computer)':ab,ti OR 'learning, machine':ab,ti OR 'transfer learning':ab,ti OR 'learning, transfer':ab,ti OR 'learning, deep':ab,ti OR 'hierarchical learning':ab,ti OR 'learning, hierarchical':ab,ti

#2

'intelligence, artificial':ab,ti OR 'computational intelligence':ab,ti OR 'intelligence, computational':ab,ti OR 'machine intelligence':ab,ti OR 'intelligence, machine':ab,ti OR 'computer reasoning':ab,ti OR 'reasoning, computer':ab,ti OR 'ai (artificial intelligence)':ab,ti OR 'computer vision systems':ab,ti OR 'computer vision system':ab,ti OR 'system, computer vision':ab,ti OR 'systems, computer vision':ab,ti OR 'vision system, computer':ab,ti OR 'vision systems, computer':ab,ti OR 'knowledge acquisition (computer)':ab,ti OR 'acquisition, knowledge (computer)':ab,ti OR 'knowledge representation (computer)':ab,ti OR 'knowledge representations (computer)':ab,ti OR 'representation, knowledge (computer)':ab,ti OR 'learning, machine':ab,ti OR 'transfer learning':ab,ti OR 'learning, transfer':ab,ti OR 'learning, deep':ab,ti OR 'hierarchical learning':ab,ti OR 'learning, hierarchical':ab,ti OR 'machine learning' OR 'artificial intelligence'
