# Supplementary material for: Diagnostic Accuracy of Artificial Intelligence Based on Imaging Data for Preoperative Prediction of Microvascular Invasion in Hepatocellular Carcinoma: A Systematic Review and Meta-Analysis
Source: Front Oncol. 2022 Feb 24;12:763842. doi: 10.3389/fonc.2022.763842 (PMC8907853; doi:10.3389/fonc.2022.763842)
Supplement: Supplementary file 3 [file Table_1.docx]

**Table S1** Baseline of the included models in this meta-analysis

| **Study** | **Model** | **MVI (+),**  **N (%)** | **HBV/HCV,**  **N (%)** | **Patient age** | **Single tumor,**  **N（%）** | **Tumor size(cm)** | **MVI (+)** | **MVI (-)** | **TP** | **FP** | **FN** | **TN** | **AUC** |
| --- | --- | --- | --- | --- | --- | --- | --- | --- | --- | --- | --- | --- | --- |
| Feng (2019)-V | NDL | 40 | 40(80.0) | 55.3 ± 12.0 | 35 (70.0) | 4.3 (2.7,6.0) | 30 | 20 | 27 | 5 | 3 | 15 | 0.833 |
| Feng (2019)-T | NDL | 38.18 | 94(85.5) | 54.6 ± 11.2 | 80 (72.7) | 3.8 (2.7,5.0) | 42 | 68 | 32 | 8 | 10 | 60 | 0.85 |
| Nebbia (2020) | NDL | 61.62 | 82(82.8) | MVI(+) 51.42±12.27  MVI(-) 54.34±9.27 | 75(75.8) | MVI(+) 7.1(3.5)  MVI(-) 6.7(3.8) | 61 | 38 | 49 | 8 | 12 | 30 | 0.867 |
| Jiang (2021)-T | DL | 54.32 | T&V:346 (85.4) | T&V:48.5±13.4 | 198(61.1) | >5cm:163,≤5cm:161 | 176 | 148 | 172 | 15 | 4 | 133 | 0.98 |
| Jiang (2021)-V | DL | 54.32 | T&V:346 (85.4) | T&V:48.5±13.4 | 48(59.3) | >5cm:39,≤5cm:42 | 44 | 37 | 41 | 9 | 3 | 28 | 0.906 |
| Wang (2020)-V | DL | 47.5 | 40(100) | MVI(+) 49.21±10.77  MVI(-) 59.14±11.04 | 29(72.5) | MVI(+) 6.4±3.5,  MVI(-) 4.4±3.4 | 19 | 21 | 14 | 4 | 5 | 17 | 0.79 |
| Wu (2021)-V | DL | 42.5 | 36(90.0) | MVI(+) 50.12±10.21  MVI(-) 52.61± 13.94 | 29(72.6) | MVI(+) 7.3±4.0 ,  MVI(-) 4.2±3.1 | 17 | 23 | 15 | 3 | 2 | 20 | 0.926 |
| Zhang (2021)-T | DL | 38.61 | T&V: 191(80.59) | MVI(+) 51(43,60)  MVI(-) 55(47,62) | 210(88.6) | MVI(+) 5.5(3.8, 9.0)  MVI(-) 3.7(2.6,6.1) | 61 | 97 | 42 | 20 | 19 | 77 | 0.81 |
| Zhang (2021)-V | DL | 39.24 | T&V: 191(80.59) | MVI(+) 51(43,60)  MVI(-) 55(47,62) | 210(88.6) | MVI(+) 5.5(3.8,9.0)  MVI(-) 3.7(2.6,6.1) | 31 | 48 | 17 | 9 | 14 | 39 | 0.72 |
| Song (2021)-V | NDL | 36.43 | 114 (81.4) | 56.66±11.92 | 140(100) | 4.80±2.92 | 51 | 89 | 33 | 28 | 18 | 61 | 0.731 |
| Song (2021)-T | NDL | 37.74 | 358 (77.7) | 56.39±11.35 | 461(100) | 5.04±3.77 | 174 | 287 | 116 | 68 | 58 | 219 | 0.764 |
| Song (2021)-V | DL | 36.43 | 114 (81.4) | 56.66±11.92 | 140(100) | 4.80±2.92 | 51 | 89 | 43 | 10 | 8 | 79 | 0.915 |
| Song (2021)-T | DL | 37.74 | 358 (77.7) | 56.39±11.35 | 461(100) | 5.04±3.77 | 174 | 287 | 147 | 46 | 27 | 241 | 0.909 |
| Liu (2021)-V | NDL | 34.43 | 56(91.8) | 52 (46,62) | 61(100) | 3.4 (2.8,4.1) | 21 | 40 | 12 | 5 | 9 | 35 | 0.745 |
| Liu (2021)-T | NDL | 33.87 | 104(83.9) | 54 (47,63) | 124(100) | 3.6 (2.8, 4.4) | 42 | 82 | 27 | 20 | 15 | 62 | 0.724 |
| Dong (2020)-V | NDL | 36.63 | T&V: 265(82.3) | MVI(-) 58±11  MVI(+) 57±9 | NA | MVI(+) 4.8±3.1  MVI(-) 3.2±2.3 | 37 | 64 | 31 | 28 | 6 | 36 | 0.726 |
| Xu (2019)-T | NDL | 28.57 | T&V: 265(82.4) | 56.6±11.4 | 402(81.2) | MVI(+) 8.7±4.2  MVI(-) 4.9±3.3 | 100 | 250 | 88 | 58 | 12 | 192 | 0.8 |
| Xu (2019)-V | NDL | 33.79 | T&V: 265(82.4) | 56.6±11.4 | 402(81.2) | MVI(+) 8.7±4.2  MVI(-) 4.9±3.3 | 49 | 96 | 44 | 20 | 5 | 76 | 0.828 |
| Yao (2018) | NDL | 48.84 | NA | NA | NA | NA | 21 | 22 | 19 | 3 | 2 | 19 | 0.98 |
| Hu (2018)-T | NDL | 39.88 | 228(66.9) | >50:198(58.1%)  <50:143(41.9%) | 341(100) | >5cm:228 (66.9%)  ≤5cm:113 (33.1%) | 136 | 205 | 88 | 47 | 48 | 158 | 0.758 |
| Hu (2018)-V | NDL | 41.84 | 124(87.9) | >50:93(66.0%)  <50:48(34.0%) | 141(100) | >5cm:60 (42.6%)  ≤5cm:81 (57.4%) | 59 | 82 | 40 | 23 | 19 | 59 | 0.731 |
| Ni (2019)-V | NDL | 39.66 | NA | MVI(+) 57±1.2  MVI(-) 59±0.8 | NA | NA | 23 | 35 | 19 | 5 | 4 | 30 | 0.88 |
| Peng (2018)-V | NDL | 61.67 | 91(75.8) | MVI(+) 55(24,73)  MVI(-) 47(19,73) | 120(100%) | MVI(+) 6.35(0.80,15.20)  MVI(-) 4.90(0.30,17.20) | 74 | 46 | 56 | 9 | 18 | 37 | 0.844 |
| Peng (2018)-T | NDL | 69.02 | 144(78.3) | MVI(+) 53(21,77)  MVI(-) 52(15,79) | 184(100%) | MVI(+) 6.30 (1.00,20.00)  MVI(-) 5.70(1.8,19.5) | 127 | 57 | 101 | 16 | 26 | 41 | 0.846 |
| Ma (2018)-V | NDL | 33.64 | 85(77.3) | MVI(+) 53(46,62)  MVI(-) 58(52,64) | 110(100%) | MVI(+) 4.9(3.6,5.9)  MVI(-) 3.7(2.6,5.0) | 37 | 73 | 28 | 9 | 9 | 64 | 0.876 |
| Ma (2018)-T | NDL | 38.3 | 34(72.3) | MVI(+) 55(34,64)  MVI(-) 59(53,63) | 47(100%) | MVI(+) 4.8(3.3,5.9)  MVI(-) 3.2(2.2,4.1) | 18 | 29 | 9 | 7 | 9 | 22 | 0.68 |
| Wei (2021)-V | DL | 46.96 | 115(100) | MVI(+) 48±11  MVI(-) 52±11 | 77(67.0%) | MVI(+) 5.8±4.3  MVI(-)4.0±3.6 | 54 | 61 | 38 | 12 | 16 | 49 | 0.802 |
| Wei (2021)-T1 | DL | 34.65 | 293(95.8) | MVI(+) 55±11  MVI(-) 59±9 | 251(77.2%) | MVI(+) 7.6±3.3  MVI(-) 4.7±2.1 | 114 | 215 | 89 | 10 | 25 | 205 | 0.798 |
| Wei (2021)-T2 | DL | 33.55 | 316(96.0) | MVI(+) 55±10  MVI(-) 54±10 | 253(76.9%) | MVI(+) 4.1±3.0  MVI(-) 2.3±1.5 | 102 | 204 | 91 | 87 | 11 | 117 | 0.93 |

NDL: non-deep learning model, DL: deep learning model, T: training set, V: validation set, Wei(2021)-V: model in validation set based on MRI, Wei(2021)-T1: model in training set based on MRI, Wei(2021)-T2:model in training set based on CT
