# Supplementary material for: Diagnostic Accuracy of Artificial Intelligence Based on Imaging Data for Preoperative Prediction of Microvascular Invasion in Hepatocellular Carcinoma: A Systematic Review and Meta-Analysis
Source: Front Oncol. 2022 Feb 24;12:763842. doi: 10.3389/fonc.2022.763842 (PMC8907853; doi:10.3389/fonc.2022.763842)
Supplement: Supplementary file 4 [file Table_2.docx]

**Table S2** Studies reporting deep learning models

| **Study** | **Research purpose** | **Method used** | **Key findings** | **Conclusions** |
| --- | --- | --- | --- | --- |
| Danjun Song (2021) | A deep learning (DL) model was developed to predict the MVI status and grade in HCC patients based on preoperative dynamic contrast-enhanced MRI and clinical parameters | The prosposed model consisted of eight CNN branches and fully connected (FC) layers and a SoftMax layer for eight MRI sequences | The DL model based only on images achieved an AUC of 0.915 in the testing cohort as compared to the radiomics model with an AUC of 0.731.Survival analysis demonstrated that the patients with DLC-predicted MVI status were associated with the poor overall survival and recurrence-free survival. | The proposed DLC model can provide a non-invasive approach to evaluate MVI before surgery, which can help surgeons make decisions of surgical strategies and assess patient’s prognosis |
| Yi‑Quan Jiang（2021） | To developed predictive models using XGBoost and deep learning based on CT images to predict MVI preoperatively. | The network takes three 16*64*64 patches from AP,PVP,DP as input and passes them through several intermediate layers to extract deep features, which are further fused and fed into the decision layers to generate the final MVI assessment result. | The AUC of the RRC Model and 3D-CNN Model in the validation set were 0.887 (95% CI 0.797–0.947) and 0.906 (95% CI 0.821–0.960), respectively (p = 0.83). Based on the MVI status predicted by the RRC and 3D-CNN Models, the mean recurrence-free survival was significantly better in the predicted MVI-negative group than that in the predicted MVI-positive group (RRC Model: 69.95 vs. 24.80 months, p < 0.001; 3D-CNN Model: 64.06 vs. 31.05 months, p = 0.027). | These non-deep learning models may facilitate decision-making in HCC treatment |
| Guangyi Wang （2020） | To investigate the value of diffusion-weighted magnetic resonance imaging for the prediction of MVI using CNN | Four CNN models consists of two convolutional layers, two max pooling layers, three fully connected layers, and a softmax layer based on b0, b100, b600, and ADC,separately. a deeply supervised net that combined the four loss functions of CNN was designed for the proposed deep learning network | Deep features in the ADC map obtained lower performance (AUC = 0.71,p= 0.012) than that of the higher b value images (b600) for MVI prediction.Furthermore, the fusion of deep features from the b0, b100,b600, and ADC images yielded the best results (AUC = 0.79, p= 0.002) for MVI prediction. | Fusion of deep features derived from DWI images concerning the three b-value images and the ADC image yields better performance for MVI prediction |
| Zhou W （2021） | To better predict MVI in HCC patients | The proposed 3D-CNN model consisted of several convolutional layers, pooling layers, fully connected layers and a softmax layer in the 3D CNN architecture.The deep supervision network (DSN) was originally proposed to directly supervise the features of the hidden layer and improve the effect of the hidden layer on the final performance during the CNN learning process. | The proposed deep learning model with deep supervision loss function produced the best results with the AUC value of 0.926 (p=0.000) | 3D CNN and deeply supervised net with contrast-enhanced MR could be effective for MVI prediction. |
| Yongxin Zhang (2021) | To develop an end-to-end deep-learning models based on MRI images for preoperative prediction of MVI | A 3D CNN was designed with nine input layers, two convolutional layers, two batchnormal layers,two fully connected layer, SoftMax layer, and output layer. Three single-layer models based on single-sequence, and fusion model combining three sequences | The fusion model achieved an area under the curve (AUC) of 0.81, sensitivity of 69%, and specificity of 79% in the training set and 0.72, sensitivity of 55%, and specificity of 81% in the validation set. | 3D CNN model may serve as a noninvasive tool to predict MVI in HCC |
| Jingwei Wei (2021) | To achieve MVI prediction via DL, which would push forward the role of noninvasive imaging in MVI preoperative anagement | Two restropective cohorts were used to train a ResNet CNN model based on CT,two restropective chorts were used to train a CNN model based on MRI.A prospective cohort was used to validate the models. | The MRI-based DL model achieved superior prediction outcome to the CT-based DL model (AUC: 0.812 vs. 0.736, p = 0.038; sensitivity:70.4% vs. 57.4%, p = 0.015; specificity: 80.3% vs. 86.9%, p = 0.052). survival analysis revealed that both DL models could stratify high and low-risk groups regarding progression free survival and overall survival (p < 0.05). | DL can be an efficient tool for MVI prediction, and EOB-MRI was proven to be the modality with advantage for MVI assessment than CE-CT |
