# Supplementary material for: Diagnostic Accuracy of Artificial Intelligence Based on Imaging Data for Preoperative Prediction of Microvascular Invasion in Hepatocellular Carcinoma: A Systematic Review and Meta-Analysis
Source: Front Oncol. 2022 Feb 24;12:763842. doi: 10.3389/fonc.2022.763842 (PMC8907853; doi:10.3389/fonc.2022.763842)
Supplement: Supplementary file 5 [file Table_3.docx]

| **Authors** | **variables with p<0.05 between MVI(+) and MVI(-)** | **variables with p<0.05 between training and texting set** |
| --- | --- | --- |
| Shi-Ting Feng (2019） | tumor size, AFP | Tumour size group（>5cm,<=5cm） |
| G. Nebbia (2020) | tumor number,Edmondson-Steiner grade | NA |
| Yidi Chen (2021) | AFP,ADC,Enhancement ratio(AP,PVP,HBP),20-min T1 relaxation time,Nonsmooth tumor margin,Peritumoral enhancement,Peritumoral hypointensity | NA |
| Peng Liu （2021） | NONE | NA |
| Yi Dong （2020） | AFP,tumor size | unclear |
| Xun Xu (2019) | clinical stage,AFP,tumor size,INR,AST | NA |
| Hangtong Hu (2018) | AFP,tumor size | NA |
| Ming Ni (2019) | none | NA |
| Jie Peng (2018) | AFP,cirrhosis,tumor margin,internal arteries,Hypoattenuating halos | none |
| Xiaohong Ma (2018) | age,afp,tumor size | none |
| Zhao Yao (2018) | none | none |
| Danjun Song (2021) | tumor size, AFP,lymphocyte counts,INR,neutrophil precentage( using the backward stepwise  method to select) | NA |
| Yi‑Quan Jiang（2021） | Lobes involved,Satellite nodule,Tumour count,Intratumour necrosis,Margin of the tumour,Internal arteries,Peritumoural enhancement,tumor size | Hypo-dense halo |
| Guangyi Wang （2020） | age,Albumin,gglutamyltransferase,tumor size,Presence of hemorrhage,Edmondson-Steiner grade | NA |
| Zhou W （2021） | age,AFP,nodules long diameter,presence of hemorrhage Edmondson-Steiner grade | NA |
| Yongxin Zhang (2021) | tumor size,Tumor pseudocapsule,Tumor stage,albumin,globulin,albumin/globulin | NA |
| Jingwei Wei (2021) | Number of tumors,Tumor size,Edmondson–Steiner grade,Peritumoral enhancement,Enhancement pattern,AFP | NA |

**Table S3** Supplementary of characteristics of the included studies

NA, not available
