# Supplementary material for: Diagnostic Accuracy of Artificial Intelligence Based on Imaging Data for Preoperative Prediction of Microvascular Invasion in Hepatocellular Carcinoma: A Systematic Review and Meta-Analysis
Source: Front Oncol. 2022 Feb 24;12:763842. doi: 10.3389/fonc.2022.763842 (PMC8907853; doi:10.3389/fonc.2022.763842)
Supplement: Supplementary file 6 [file Table_4.docx]

**Table S4** Meta-regression analysis for NDL and DL models, excluding models using ultrasound

| **Parameter** | **Category** | **Studies,**  **N** | **Univariate meta-reg.** | | | | **Multivariate meta-reg.** |
| --- | --- | --- | --- | --- | --- | --- | --- |
|  |  |  | **Sensitivity** | **p** | **Specificity** | **p** | **p** |
| image | MRI | 13 | 0.78[0.70-0.85] | **0.00** | 0.82[0.78-0.87] | **0.00** | 0.33 |
|  | CT | 12 | 0.83[0.77-0.89] |  | 0.79[0.74-0.84] |  |  |
| number | solitary | 10 | 0.72[0.64-0.81] | **0.00** | 0.80[0.75-0.86] | **0.00** | **0.05** |
|  | multiple | 15 | 0.85[0.80-0.90] |  | 0.81[0.77-0.86] |  |  |
| ap | Yes | 21 | 0.82[0.77-0.87] | 0.59 | 0.81[0.77-0.85] | **0.00** | 0.35 |
|  | No | 4 | 0.71[0.54-0.87] |  | 0.82[0.73-0.91] |  |  |
| pvp | Yes | 20 | 0.81[0.75-0.86] | 0.12 | 0.81[0.77-0.85] | **0.00** | 0.85 |
|  | No | 5 | 0.77[0.65-0.90] |  | 0.80[0.71-0.89] |  |  |
| segment | Semiautomatically | 4 | 0.84[0.74-0.94] | 0.13 | 0.77[0.68-0.87] | **0.00** | 0.48 |
|  | manual | 21 | 0.79[0.74-0.85] |  | 0.82[0.78-0.85] |  |  |
| lasso | Yes | 8 | 0.75[0.65-0.85] | **0.00** | 0.77[0.70-0.84] | **0.00** | 0.13 |
|  | No | 17 | 0.83[0.77-0.88] |  | 0.83[0.78-0.87] |  |  |
| svm | Yes | 6 | 0.81[0.70-0.91] | **0.03** | 0.81[0.74-0.88] | **0.00** | 0.99 |
|  | No | 19 | 0.80[0.74-0.86] |  | 0.81[0.77-0.85] |  |  |
| cnn | Yes | 6 | 0.81[0.72-0.91] | **0.03** | 0.83[0.77-0.90] | **0.00** | 0.68 |
|  | No | 19 | 0.80[0.74-0.86] |  | 0.80[0.76-0.84] |  |  |
| 3dcnn | Yes | 5 | 0.86[0.77-0.95] | 0.17 | 0.84[0.76-0.91] | **0.00** | 0.39 |
|  | No | 20 | 0.79[0.73-0.84] |  | 0.80[0.76-0.84] |  |  |
| set | Yes | 13 | 0.79[0.71-0.86] | **0.00** | 0.82[0.77-0.87] | **0.00** | 0.71 |
|  | No | 12 | 0.81[0.75-0.88] |  | 0.80[0.75-0.85] |  |  |
