# Supplementary material for: Diagnostic Accuracy of Artificial Intelligence Based on Imaging Data for Preoperative Prediction of Microvascular Invasion in Hepatocellular Carcinoma: A Systematic Review and Meta-Analysis
Source: Front Oncol. 2022 Feb 24;12:763842. doi: 10.3389/fonc.2022.763842 (PMC8907853; doi:10.3389/fonc.2022.763842)
Supplement: Supplementary file 7 [file Table_5.docx]

| **Parameter** | **Category** | **Univariate meta reg.** | | | | | **Multivariate meta-reg.** |
| --- | --- | --- | --- | --- | --- | --- | --- |
|  |  | studies,n | Sensitivity | p | Specificity | p | p |
| image | MRI | 5 | 0.79[0.69-0.88] | **0.05** | 0.75[0.70-0.79] | **0.00** | 0.28 |
|  | CT | 9 | 0.76[0.69-0.84] | . | 0.79[0.76-0.82] | . |  |
| number | Yes | 8 | 0.70[0.65-0.75] | **0.00** | 0.77[0.74-0.80] | **0.00** | **0.00** |
|  | No | 6 | 0.87 [0.83-0.91] | . | 0.78[0.74-0.82] | . |  |
| segment | Yes | 4 | 0.84[0.77-0.91] | 0.10 | 0.77[0.72-0.81] | **0.00** | 0.15 |
|  | No | 10 | 0.74[0.67-0.81] | . | 0.78[0.75-0.82] | . |  |
| lasso | Yes | 6 | 0.78[0.69-0.87] | **0.02** | 0.77[0.73-0.82] | **0.00** | 0.99 |
|  | No | 8 | 0.77[0.69-0.85] | . | 0.77[0.75-0.80] | . |  |
| svm | Yes | 8 | 0.77[0.69-0.85] | **0.01** | 0.77[0.75-0.80] | **0.00** | 0.99 |
|  | No | 6 | 0.78[0.69-0.87] | . | 0.77[0.73-0.82] | . |  |
| set | Yes | 7 | 0.78[0.70-0.87] | **0.03** | 0.80[0.76-0.84] | **0.00** | 0.39 |
|  | No | 7 | 0.77[0.69-0.85] | . | 0.76[0.73-0.79] | . |  |

**Table S5** Meta-regression analysis for NDL models, excluding models using ultrasound
